# The Slow Oscillation in Cortical and Thalamic Networks: Mechanisms and Functions

**DOI:** 10.3389/fncir.2015.00088

**Published:** 2016-01-14

**Authors:** Garrett T. Neske

**Affiliations:** ^1^Department of Neuroscience, Division of Biology and Medicine, Brown UniversityProvidence, RI, USA; ^2^Department of Neurobiology, Yale UniversityNew Haven, CT, USA

**Keywords:** slow oscillation, Up state, cortex, thalamus, sleep

## Abstract

During even the most quiescent behavioral periods, the cortex and thalamus express rich spontaneous activity in the form of slow (<1 Hz), synchronous network state transitions. Throughout this so-called slow oscillation, cortical and thalamic neurons fluctuate between periods of intense synaptic activity (Up states) and almost complete silence (Down states). The two decades since the original characterization of the slow oscillation in the cortex and thalamus have seen considerable advances in deciphering the cellular and network mechanisms associated with this pervasive phenomenon. There are, nevertheless, many questions regarding the slow oscillation that await more thorough illumination, particularly the mechanisms by which Up states initiate and terminate, the functional role of the rhythmic activity cycles in unconscious or minimally conscious states, and the precise relation between Up states and the activated states associated with waking behavior. Given the substantial advances in multineuronal recording and imaging methods in both *in vivo* and *in vitro* preparations, the time is ripe to take stock of our current understanding of the slow oscillation and pave the way for future investigations of its mechanisms and functions. My aim in this Review is to provide a comprehensive account of the mechanisms and functions of the slow oscillation, and to suggest avenues for further exploration.

## Introduction

The mammalian neocortex is a massively interconnected synaptic network. The vast majority of excitatory synapses onto cortical excitatory neurons come from other cortical excitatory neurons (Braitenburg and Shüz, 1998; Binzegger et al., 2004; Douglas and Martin, 2004). One consequence of the vast recurrent connectivity of the neocortex is the ability to initiate and sustain patterned network activity, even in the virtual absence of sensory stimulation, such as during quiescent sleep and anesthesia. During these quiescent periods, the entire neocortex undergoes slow, synchronized transitions between vigorous synaptic activity (Up states) and relative silence (Down states). This cycling (<~1 Hz) between Up and Down states constitutes the *slow oscillation*.

The occurrence of the slow oscillation has been well-documented from intracellular and extracellular recording and imaging in various experimental preparations, including anesthetized, naturally sleeping, and quiescent waking animals. Great strides have been made in uncovering the cellular and network mechanisms involved in this widespread phenomenon. Several mechanistic features of the slow oscillation, however, remain to be explored, especially the initiation and termination of Up states and the roles of subcortical structures in sustaining and pacing the slow oscillation in the cortex. Furthermore, very little is known about the functional roles of the slow oscillation and the exact relation between Up states and activated states of the awake cortex.

In this Review, I provide a comprehensive account of the mechanisms and functions of the slow oscillation in the cortex and thalamus and also indicate areas requiring further investigation. I first consider the phenomenology of the slow oscillation in the cortex and thalamus. I then discuss the cellular and network mechanisms thought to be involved in the initiation, persistence, and termination of Up states. I then consider the involvement of subcortical structures in either modulating or mediating the slow oscillation in cortex. In the latter half of the Review, I discuss the putative functional roles of the slow oscillation, concluding with a consideration of how Up states may be a manifestation of dynamic routing of information flow in cortical networks.

## The Discovery of the Slow Oscillation and Its Characterization

**Figure 1:**
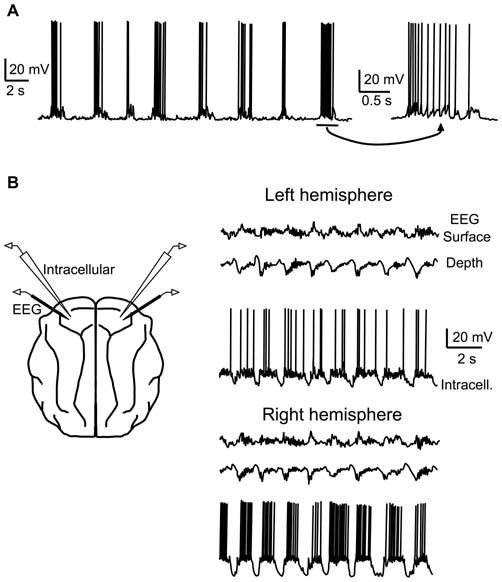
**Early recordings of the cortical slow oscillation in anesthetized cats. (A)** Intracellular recording from a putative pyramidal cell in cat suprasylvian area five engaged in the slow oscillation under urethane anesthesia. **(B)** Dual intracellular recordings of putative pyramidal cells during slow oscillation in left (top) and right (bottom) hemispheres of motor cortex in ketamine-xylazine-anesthetized cat. Surface and depth EEG are also recorded in each hemisphere. Cells in both hemispheres are coherent with the population oscillation. **(A)** adapted by permission from the Society for Neuroscience: *Journal of Neuroscience* (Steriade et al., 1993a), © 1993. **(B)** adapted by permission from the Society for Neuroscience: *Journal of Neuroscience* (Contreras and Steriade, 1995), © 1995.

While first described in the striatum of anesthetized rats (Wilson and Groves, 1981), in a series of three articles published in the *Journal of Neuroscience* in 1993, Steriade et al. (1993a,b,c) provided the first characterization of the slow oscillation in cortical and thalamic networks using intracellular and EEG recordings in anesthetized cats. During the slow oscillation, most neurons showed periods of suprathreshold depolarization, interspersed with periods of relative inactivity (“Up” and “Down” states in the later literature, respectively; Figure 1A). The depolarizing periods were associated with barrages of synaptic inputs, while the silent periods showed a marked withdrawal of these inputs. Importantly, the cortical slow oscillation persisted after thalamic and callosal lesions (Steriade et al., 1993b), suggesting that the cortical network itself is sufficient for the generation of the oscillation (but see “Contribution of the thalamus” Section). Later work demonstrated that the slow oscillation could also be expressed in deafferented cortical slabs of a certain size (Timofeev et al., 2000), as well as in cortical slice preparations *in vitro* under certain conditions (Sanchez-Vives and McCormick, 2000; see “The slow oscillation *in vitro*” Section).

The slow oscillation is a global and synchronized network phenomenon, engaging neurons throughout the cortex (Figure 1B), and also involving neurons in several subcortical areas, including the thalamus (see “Contribution of the thalamus” Section), striatum (see above), and the cerebellum (Ros et al., 2009). Within the local cortical network (within a few tens of millimeters), cortical neurons synchronously depolarize and hyperpolarize during the slow oscillation, with phase delays less than an order of magnitude of the oscillation period (<100 ms; Amzica and Steriade, 1995a; Volgushev et al., 2006). The long-range coherency of the slow oscillation likely depends upon horizontal axon collaterals of cortical pyramidal cells (Amzica and Steriade, 1995b), though diffusely projecting thalamocortical neurons from higher-order and intralaminar thalamic nuclei may also play a role in synchronizing the cortical population (Sheroziya and Timofeev, 2014; see also “Contribution of the thalamus” Section).

The spatiotemporal evolution of the slow oscillation often exhibits greater complexity than a simultaneous activation of all neurons in the local cortical network. Recordings from high-density EEG (Massimini et al., 2004) and extracellular arrays (Luczak et al., 2007) indicate that the slow oscillation *in vivo* propagates as a traveling wave, often in the anteroposterior direction. The slow oscillation also activates neurons in particular, stereotyped sequences (Luczak et al., 2007). The complex spatiotemporal architecture of the slow oscillation may provide a mechanism for essential computations during slow-wave sleep, such as those related to memory consolidation (see also “Synaptic plasticity and the slow oscillation” Section).

## The Slow Oscillation *In Vitro*

The genesis of the slow oscillation seems largely endemic to cortex, since, as discussed in the previous section, the oscillation can persist in the absence of thalamic input. The extent of the ability of the local cortical network to generate slow oscillations is perhaps most exemplified by the fact the cortical slice preparation *in vitro* can also exhibit this activity.

**Figure 2:**
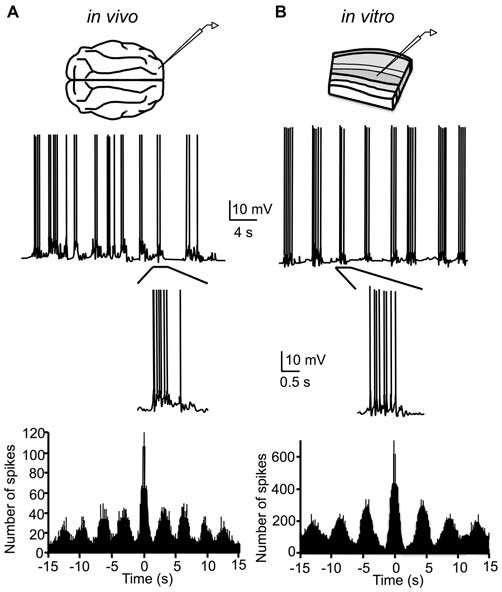
**Original demonstration of the slow oscillation in cortical slices. (A)** Intracellular recording of cortical neuron in cat primary visual cortex during the slow oscillation under halothane anesthesia. Rhythmicity is quantified by the autocorrelogram of the intracellular record (bottom). **(B)** Intracellular recording of cortical neuron of ferret visual cortex *in vitro* in “modified ACSF.” The slow oscillation *in vitro* is also rhythmic (bottom), though with a longer oscillation period than the *in vivo* slow oscillation. **(A,B)** adapted by permission from Macmillan Publishers Ltd: *Nature Neuroscience* (Sanchez-Vives and McCormick, 2000), © 2000.

In the process of determining why short-term synaptic depression is often higher *in vitro* compared to *in vivo* (Sanchez-Vives, 2007), Sanchez-Vives and McCormick (2000) discovered that by slightly modifying the ionic composition of the artificial cerebrospinal fluid (ACSF) bathing slices of ferret visual and prefrontal cortex, rhythmic spontaneous network activity occurring at ~0.3 Hz could be recorded both intracellularly and in the multi-unit activity. Specifically, by reducing the concentrations of Mg^2+^ and Ca^2+^ from (in mM) 2 and 2–1 and 1.2, respectively, and increasing the concentration of K^+^ from 2.5–3.5, slow oscillatory activity arose in the slice that was largely indistinguishable from the slow oscillation *in vivo*, albeit with a lower frequency (Figure 2). Notably, the reduced Mg^2+^ and Ca^2+^ concentrations are actually closer to those measured *in situ* (Somjen, 2004). The effect of these changes in ionic concentrations is to increase the overall excitability of neurons, either through direct depolarization of the resting membrane potential or through shifts in the activation curves of various voltage-dependent conductances. The increased K^+^ concentration presumably depolarizes the resting membrane potential via a less negative K^+^ Nernst potential; the resting neuronal membrane is primarily permeable to K^+^. The reduced concentration of Mg^2+^ and Ca^2+^ may enhance excitability through reduced charge screening of the neuronal membrane, resulting in a negative shift in the activation curves for voltage-dependent conductances (Frankenhaeuser and Hodgkin, 1957; McLaughlin et al., 1971). These ionic concentrations afford a critical level of excitability in the *in vitro* recurrent cortical network such that reverberant activity can be sustained in rhythmic cycles.

The ability of relatively small cortical circuits to exhibit the slow oscillation seems to be a generalizable feature across cortical areas and species. After the slow oscillation was first demonstrated in ferret visual and prefrontal cortex *in vitro*, various groups showed that similar behavior could be expressed in slices from other cortical areas and species, including: ferret piriform cortex (Sanchez-Vives et al., 2008), mouse barrel cortex (MacLean et al., 2005; Rigas and Castro-Alamancos, 2007; Watson et al., 2008; Rigas and Castro-Alamancos, 2009; Fanselow and Connors, 2010; Yassin et al., 2010; Favero et al., 2012; Favero and Castro-Alamancos, 2013; Sippy and Yuste, 2013; Neske et al., 2015), rat barrel cortex (Wester and Contreras, 2012, 2013), mouse entorhinal cortex (Tahvildari et al., 2012; Craig et al., 2013; Neske et al., 2015; Salkoff et al., 2015), rat entorhinal cortex (Cunningham et al., 2006; Mann et al., 2009; Mayne et al., 2013), and rat prefrontal cortex (Wang et al., 2010).

Acute cortical slice preparations provide numerous experimental benefits, such as control of the composition of the extracellular environment and ease of recording and imaging from particular neuron types throughout all layers of cortex. These benefits have been harnessed to study the slow oscillation in exquisite detail, revealing the synaptic and intrinsic currents involved in the spatiotemporal evolution of network activity. Intracellular and extracellular recording, as well as calcium and voltage-sensitive dye imaging of cortical slices exhibiting the slow oscillation have provided important details about the cellular and network mechanisms involved, especially when combined with pharmacological isolation of particular conductances.

## Cellular and Network Mechanisms of the Slow Oscillation

The entire cortical network is engaged by the slow oscillation. While it seems almost inevitable that such a highly interconnected system as the cortex would generate such activity, there are many aspects of the slow oscillation that deserve attention. How does it initiate? How does it persist? What terminates it? Why is it rhythmic? Such questions concerning the cellular and network mechanisms of the slow oscillation arose even from the very first observation of its presence in the cortex by Steriade and colleagues (see “The discovery of the slow oscillation and its characterization” Section). Since that time, considerable progress has been made toward a detailed understanding of the slow oscillation.

### Up State Initiation

How does synchronous network activation arise from a relatively silent network? Current thought about the initiation of cortex-wide activity during the slow oscillation is largely divided between two mechanisms: initiation by persistently active, pacemaker-like cortical neurons and stochastic initiation by temporal summation of spontaneous synaptic activity. In both cases, pyramidal cells in cortical layer 5 are considered to be the key players.

There is substantial evidence that cortical layer 5 is instrumental in the initiation of Up states. Early current-source-density analysis *in vivo* revealed that the polarity of the extracellular potential recorded during the slow oscillation reversed from source to sink toward the middle cortical layers (Steriade and Amzica, 1996). The first *in vitro* recordings of the slow oscillation (see “The slow oscillation *in vitro*” Section) showed that multi-unit activity was strongest and earliest in layer 5 (Sanchez-Vives and McCormick, 2000). Furthermore, when synaptic connections between the upper and lower layers of cortex were severed via a horizontal cut through layer 4, the lower cortical layers could still generate the slow oscillation, while the upper cortical layers could generate this activity either infrequently or not at all. The importance of the layer 5 cortical network in generating Up states has also been demonstrated in later *in vitro* work (Wester and Contreras, 2012) and through optogenetic manipulation of layer 5 and layer 2/3 pyramidal cells *in vivo* (Beltramo et al., 2013).

There are several possible reasons why layer 5 is critical for the initiation of the slow oscillation. Many layer 5 pyramidal cells exhibit intrinsic rhythmicity, with resonant firing frequencies <15 Hz following short depolarizing or hyperpolarizing current pulses (Agmon and Connors, 1989; Silva et al., 1991). Such resonance at low frequencies could facilitate the emergence of the slow oscillation in the entire cortical network. Within layer 5, there is also a subtype of pyramidal cell that displays bursts of action potentials upon depolarizing current injection (Connors et al., 1982). This subtype of pyramidal cell was originally suggested to play the dominant role in the initiation of epileptiform activity in the cortex (Connors, 1984; Chagnac-Amitai and Connors, 1989). In addition to signaling through bursts of action potentials, these pyramidal cells have other features that are conducive to initiating network activity, such as wide axonal arborization within layer 5 (Chagnac-Amitai et al., 1990) and high spine density on their dendrites (DeFelipe and Fariñas, 1992), implying an especially high degree of divergence and convergence from and onto these cells. As a consequence of this synaptic architecture, horizontal propagation of epileptiform discharges occurs preferentially via pathways in layer 5 (Telfeian and Connors, 1998; Connors et al., 2001; Pinto et al., 2005). Comparably to their putative role in the initiation of seizure-like activity, intrinsic bursting pyramidal cells in layer 5 may also play a role in the initiation of Up states (Lörincz et al., 2015). Indeed, it could be argued that the paroxysmal activity associated with certain epileptic seizures are actually dysregulated Up states (Žiburkus et al., 2013). Consistent with this proposed role for intrinsic-bursting pyramidal cells in Up state initiation are intracellular recordings during the slow oscillation in anesthetized and sleeping cats, showing that these cells often fire before all other recorded neuron types preceding Up state onset (Chauvette et al., 2010).

The intrinsic and synaptic properties of pyramidal cells in layer 5 establish these cells as well-primed to initiate the Up states of the slow oscillation, but how does activity emerge in these cells in the first place during the quiescent Down state? There are at least two plausible scenarios for the intracortical origination of the Up state.

In one scenario, spontaneous, action potential-independent excitatory synaptic potentials (i.e., miniature EPSPs; Fatt and Katz, 1952) temporally summate in a critical number of layer 5 pyramidal neurons, driving these neurons to spike. These neurons then tip the entire cortical network into the Up state. Evidence for this hypothesis of Up state generation comes from both computational models (Timofeev et al., 2000; Bazhenov et al., 2002) and intracellular recordings during the slow oscillation in sleeping and anesthetized cats (Chauvette et al., 2010). In the latter study, cells that were active earliest in the Down to Up state transition exhibited a slow ramp of depolarization crowned by putative EPSPs, whereas cells active later in the transition depolarized more rapidly, without the presence of visibly discrete EPSPs.

In another scenario for Up state generation, layer 5 pyramidal cells that fire persistently during the Down state initiate the Up state after the cellular and network refractory mechanisms associated with the previous Up state have subsided (see “Up state termination” Section). The observation of persistently firing layer 5 pyramidal cells during the Down state is variable in the literature. Neither intracellular nor extracellular recordings in most of the studies of the slow oscillation in anesthetized and sleeping cats have shown the presence of spiking activity during the Down state in any layer of cortex. During slow oscillations *in vitro*, however, pyramidal cells in layer 5 often exhibit spontaneous firing during the Down state (Sanchez-Vives and McCormick, 2000), many of which might be of the intrinsic-bursting subtype (Neske et al., 2015). As expected due to its occurrence during the Down state, the spontaneous firing of these layer 5 pyramidal cells seems to be independent of synaptic activity, since it persists under blockade of fast excitatory and inhibitory synaptic transmission (Le Bon-Jego and Yuste, 2007). The spontaneous firing of certain layer 5 pyramidal neurons during the Down state, or at least the presence of a signal in the multi-unit activity, has also been reported during the slow oscillation in rodents *in vivo* (Hasenstaub et al., 2007; Sakata and Harris, 2009; Crunelli et al., 2012).

The oscillation period of the slow oscillation depends upon the interplay between Up state initiation mechanisms and the refractory mechanisms associated with the Down state (see “Up state termination” Section), with a possible contribution from the intrinsic rhythmicity of certain layer 5 pyramidal cells (Lörincz et al., 2015). After an Up state terminates, a sufficient amount of synaptic activity, either due to action-potential-independent synaptic release or persistently firing pyramidal cells, must accumulate in the network to ignite the next Up state. The potential for synaptic activity during the Down state to trigger another Up state depends first and foremost on when this activity occurs during the network refractory period, which is most likely set by the level of activation and inactivation of activity-dependent K^+^ conductances that were opened during the Up state (see “Up state termination” Section). Akin to the absolute and relative refractory periods associated with single action potentials, as well as epileptiform discharges (Gutnick et al., 1982), there appears to be a time period following an Up state during which another Up state cannot be elicited (Sanchez-Vives and McCormick, 2000). This “absolute” network refractory period sets a lower bound on the oscillation period of the slow oscillation. While “absolute” in the sense that an Up state cannot be elicited even with a high degree of synaptic activity (e.g., via electrical stimulation or glutamate puff), this refractory period is likely regulable to the extent that activity-dependent K^+^ conductances are regulable (e.g., via neuromodulatory tone). The next factor that determines the slow oscillation period is the level of synaptic activity during the Down state. If the amount of synaptic activity exceeds some critical threshold after enough K^+^ conductances have inactivated, another Up state will initiate. As discussed above, the origin of this synaptic activity may be either the persistent firing of certain layer 5 pyramidal cells, miniature EPSPs, or both. At the most basic level, the amount of synaptic activity contributed by either of these sources will depend upon the size of the network; with more synapses impinging on a given post-synaptic cell, the higher the probability of EPSPs (miniature or evoked) temporally summating to a critical level to initiate an Up state. This is presumably the major reason why the slow oscillation often has a lower frequency *in vitro* than *in vivo*, in which in the former case, the number of synapses is significantly truncated. The amount of synaptic activity during the Down state likely has multiple points of regulation, and consequently multiple mechanisms by which the period of the slow oscillation can be adjusted. If Down-state synaptic activity depends significantly on the persistent firing of certain layer 5 pyramidal cells, this property may be subject to multiple types of neuromodulatory control (Dembrow and Johnston, 2014; see also “Contribution of the basal forebrain and brainstem nuclei” Section). If the temporal summation of miniature EPSPs is the driver of Up state initiation, there are multiple mechanisms for the regulation of this process as well, many of which affect either miniature EPSP size or frequency independently of action-potential-dependent synaptic release (Ramirez and Kavalali, 2011).

Another issue regarding Up state initiation is the fine-scale specificity of the neuronal sub-networks that first engage the rest of the network: can an Up state in principle initiate in any sub-network or does the same sub-network initiate the Up state every time in a given cortex, whether slice, slab, or intact brain? Evidence from calcium imaging in cortical slices (Mao et al., 2001; Cossart et al., 2003; Ikegaya et al., 2004; MacLean et al., 2005) and laminar extracellular probes *in vivo* (Luczak et al., 2007) suggest that Up states initiate in the same groups of neurons and engage the rest of the cortical network in the same sequence on each cycle. Occasionally, however, Up states can initiate in different sequences of cells or travel in different directions either spontaneously or due to stimulation of a region distinct from the “default” region of Up state initiation (Sanchez-Vives and McCormick, 2000; Luczak et al., 2007). Thus, while Up state initiation appears to depend upon the stereotypical activation of specific sub-networks, this is likely not an immutable property.

### Up State Persistence

Up states of the slow oscillation are persistent network events, sustained for hundreds of milliseconds to a few seconds. Persistent action potential output in neurons in the absence of a stimulus or following the termination of a stimulus is a prevalent phenomenon in most central nervous system (CNS) structures. One important mechanistic issue concerning persistent neuronal activity is the relative contribution of synaptic vs. intrinsic membrane properties in sustaining such activity (Marder et al., 1996; Major and Tank, 2004). Purely intrinsic mechanisms for bi- or multistability of neuronal activity have been documented for certain cells under certain conditions in the mammalian CNS. Motor neurons of the spinal cord *in vitro* exhibit intrinsic persistent activity in the form of plateau potentials mediated by L-type Ca^2+^ channels (Alaburda et al., 2002). Plateau potentials are also a prominent feature of cerebellar Purkinje cells *in vitro* (Llinás and Sugimori, 1980) and *in vivo* (Loewenstein et al., 2005). Persistent neuronal activity due to intrinsic membrane mechanisms has been demonstrated to a lesser degree in cerebral cortex, though there are some notable examples. Pyramidal cells of the entorhinal cortex *in vitro* in the presence of carbachol exhibit graded persistent activity, in which depolarizing intracellular current stimuli lead to progressively more intense persistent firing outlasting the stimulus (Egorov et al., 2002; Fransén et al., 2006). This phenomenon may depend upon Ca^2+^-dependent nonspecific cation currents (I_CAN_).

Does activity during the Up state persist primarily as a consequence of cell-intrinsic mechanisms or synaptic mechanisms? While a certain role for intrinsic mechanisms cannot be entirely ruled out (particularly in the layer 5 pyramidal cells discussed earlier), most evidence suggests that persistent activity during the Up state crucially depends on recurrent excitatory synaptic activity, balanced by synaptic inhibition. Several features of the Up state from intracellular recordings suggest a predominately synaptic basis for persistent activity. First, injection of current to either depolarize or hyperpolarize the membrane potential of recorded cells does not affect the duration of the Up state or its rhythmicity (Steriade et al., 1993a; Contreras et al., 1996b; Sanchez-Vives and McCormick, 2000; McCormick et al., 2003; Shu et al., 2003a), contrary to the expectation if voltage-dependent conductances were involved. Second, both membrane potential variance and irregularity of interspike intervals are high in cortical neurons during Up states, consistent with a role of excitatory and inhibitory synaptic barrages sustaining persistent activity. Third, Up states are completely abolished in cortical slices during application of antagonists of fast glutamatergic transmission (i.e., α-amino-3-hydroxy-5-methyl-4-isoxazolepropionic acid, AMPA or N-Methyl-D-aspartate, NMDA receptor antagonists; Sanchez-Vives and McCormick, 2000; McCormick et al., 2003; Shu et al., 2003a). Regarding the last point, while both AMPA and NMDA receptor activation appear to be necessary for the natural persistence of the Up state, NMDA receptors may play a paramount role (Major et al., 2013). Blockade of NMDA receptors by supplemental doses of ketamine to pre-existing urethane anesthesia greatly reduced Up state duration (by more than half) in the original *in vivo* characterization of the cortical slow oscillation (Steriade et al., 1993a). Additionally, bath-application of NMDA receptor antagonists in slices exhibiting the slow oscillation virtually abolishes this activity (Sanchez-Vives and McCormick, 2000; Favero and Castro-Alamancos, 2013; Castro-Alamancos and Favero, 2015), while bath-application of AMPA receptor antagonists can unmask Up states that are entirely dependent on NMDA receptors for fast glutamatergic signaling (Favero and Castro-Alamancos, 2013). The role of NMDA receptors in the persistence of the Up state is evocative of its purported role in the persistent activity underlying working memory in prefrontal cortex (Lisman et al., 1998; Wang et al., 2013). Activation of NMDA receptors also sustains certain paroxysmal oscillations originating in layer 5 (Silva et al., 1991; Flint and Connors, 1996). Interestingly, in rat entorhinal cortex *in vitro*, only selective blockade of kainate receptors abolished the slow oscillation (Cunningham et al., 2006).

**Figure 3:**
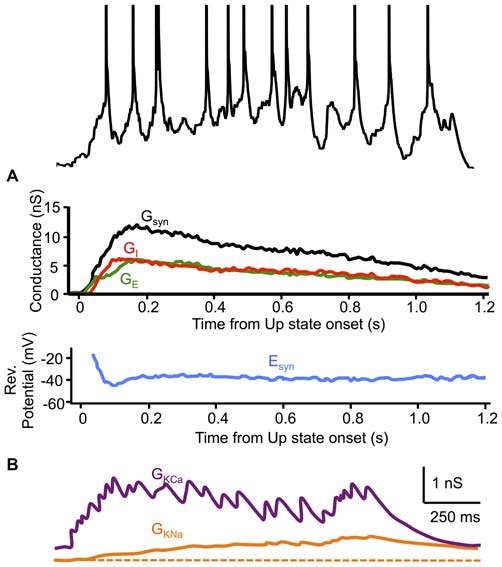
**Dynamics of synaptic and intrinsic conductances during Up states. (A)** Excitatory and inhibitory synaptic conductances (G_E_ and G_I_, respectively) (G_syn_ = G_E_ + G_I_) decrease proportionally throughout the Up state, maintaining a relatively constant synaptic reversal potential (E_syn_). Conductance estimates are from voltage-clamp recordings in cortical neurons of ferret visual or prefrontal cortex *in vitro*. **(B)** Two activity-dependent K^+^ conductances (G_KCa_ and G_KNa_) are activated during Up states in a model recurrent cortical network. **(A)** adapted by permission from Macmillan Publishers Ltd: *Nature* (Shu et al., 2003b), © 2003. **(B)** adapted by permission from the American Physiological Society: *Journal of Neurophysiology* (Compte et al., 2003), © 2003.

Synaptic inhibition also plays a critical role in the sustenance of Up state activity by maintaining the membrane potential at a level near spike threshold where synaptic noise can transiently cause firing. Neurons secure this stable region of membrane potential dynamics as a consequence of a precise balance between synaptic excitation and inhibition. During Up states in ferret cortex *in vitro* (Shu et al., 2003b) and *in vivo* (Haider et al., 2006), excitatory and inhibitory conductances change proportionally, often with an approximately unity ratio, at least as measured from the soma (Figure 3A). The result of this balance is that, even as the total synaptic conductance changes during the Up state, the synaptic reversal potential remains essentially fixed, supporting a steady level of the membrane potential near spike threshold (≃37 mV). During Up states in other cortical areas and other species, while excitatory and inhibitory conductances vary mostly concomitantly, there is divergence in both the reported ratios of these conductances and their precise time-courses. As mentioned above, estimated excitatory and inhibitory conductances in the deep layers of ferret prefrontal and visual cortex *in vitro* (Shu et al., 2003b) and the deep layers of prefrontal cortex in ketamine-xylazine-anesthetized ferrets (Haider et al., 2006) were markedly similar in magnitude throughout the duration of the Up state, with the exception of the very beginning and end of the Up state, where excitation dominated. In the deep layers of parietal association cortex of naturally sleeping cats, however, the estimated inhibitory conductance strongly dominated the excitatory conductance, by ~10:1, throughout the majority of the Up state (Rudolph et al., 2007). While not as pronounced as the latter study, inhibition also dominated excitation, by ~2:1, in the estimated synaptic conductance during Up states in both the deep and superficial layers of mouse barrel cortex *in vitro*, though primarily during the first half of the Up state (Neske et al., 2015). In one study of the superficial layers of barrel cortex of urethane-anesthetized rats, Up states were overwhelmingly dominated by excitation, with an estimated excitatory-to-inhibitory conductance ratio of ~10:1 (Waters and Helmchen, 2006).

### Up State Termination

The central contention regarding the termination of Up states is its dependance on synaptic vs. intrinsic cellular mechanisms, notably a similar debate with regard to the termination of seizure activity (McCormick and Contreras, 2001). More specifically, Up state termination may result either from enhanced activity of inhibitory interneurons near the end of the Up state (or synaptic depression of excitatory synapses) or from the activation of activity-dependent hyperpolarizing conductances. Of course, intrinsic and synaptic mechanisms of Up state termination are likely not mutually exclusive. In principle, any of these mechanisms would lead to disfacilitation (i.e., removal of synaptic inputs) in excitatory cells and an eventual failure of the network’s ability to sustain the Up state.

In the original characterization of the cortical slow oscillation (Steriade et al., 1993a), the authors considered Ca^2+^-dependent K^+^ (K_Ca_) conductances as candidates for the termination of Up states based upon the long-lasting afterhyperpolarizations (AHPs) they effect in pyramidal cells (Schwindt et al., 1992). Shortly after the initial characterization of the cortical slow oscillation, Steriade et al. (1993d) showed that Up-Down transitions were abolished by stimulation of brainstem nuclei originating cholinergic projections to cortex. This effect was dependent on muscarinic, but not nicotinic signaling. These results suggested that activity-dependent K^+^ channels, such as K_Ca_, might be crucial for the termination of Up states, since such channels are blocked by acetylcholine via muscarinic receptors (McCormick and Williamson, 1989). Both experimental (Sanchez-Vives and McCormick, 2000) and computational modeling studies (Compte et al., 2003) have suggested that activation of Na^+^-dependent K^+^ (K_Na_) conductances may also play a role in the termination of Up states since these conductances also cause an activity-dependent slow AHP in cortical neurons, which is also blocked by acetylcholine (Schwindt et al., 1989; Sanchez-Vives et al., 2000). Additional K^+^ conductances that may play a role in Up state termination include those activated by extracellular adenosine (Phillis et al., 1975) or intracellular ATP (Ashford et al., 1988), whose concentrations increase with enhanced spiking activity due to the increased metabolic load of synchronous network activity. The role of increased extracellular adenosine in terminating Up states through blockade of a K^+^ conductance has been hypothesized (Steriade et al., 1994; Amzica and Steriade, 1995a; Contreras and Steriade, 1995; Contreras et al., 1996b), while the role of K^+^ conductances activated by increased intracellular ATP in Up state termination was suggested by the increased duration of cortical Up states *in vitro* under non-specific pharmacological blockade of these conductances (Cunningham et al., 2006).

Activation of activity-dependent K^+^ conductances during Up states would decrease the excitability of neurons in which these conductances are expressed (i.e., predominately cortical pyramidal cells), decreasing their firing rates, thus leading to a generalized disfacilitation in cortical networks and Up state termination (Figure 3B). Disfacilitation can also result from short-term synaptic depression, a feature of intracortical excitatory synapses (Thomson and Deuchars, 1994). The relative importance of synaptic depression vs. the activation of K^+^ conductances for disfacilitation during the Up state is unresolved. Measurements of input resistance of neurons during the slow oscillation show a minimum at the beginning of the Up state, which steadily increases toward a maximum at the point of Up state termination (Contreras et al., 1996b). This temporal profile of input resistance is consistent with a role for synaptic depression in terminating the Up state, since a role for the activation of K^+^ conductances might predict a decrease in input resistance as the Up state progresses. Changes in input resistance, however, would be due to both the closing of synaptic conductances and the opening of K^+^ conductances. If the synaptic conductance decrease is greater than the K^+^ conductance increase, then the overall effect would be an increase in input resistance, though the K^+^ conductance increase might still be contributing via hyperpolarization. Indeed, it might be this hyperpolarizing effect that contributes to decreased firing in excitatory cells, leading to decreased synaptic conductance. Thus, it is difficult to determine from input resistance changes alone whether synaptic depression or K^+^ conductance increase is the primary cause of Up state termination. Nevertheless, if activity-dependent K^+^ conductances contribute to Up state termination, a prediction might be that a slight decrease in input resistance occurs after the synaptic conductance decays completely at the end of the Up state but while the K^+^ conductance is still active. Input resistance measurements during the Down state in Sanchez-Vives and McCormick (2000) are consistent with this notion, as is the temporal profile of input resistance in a cortical network model of the slow oscillation (Compte et al., 2003), though this particular model did not include synaptic depression. One model including both activity-dependent K^+^ conductances and synaptic depression concluded that both are important for Up state termination, but did not manipulate either variable alone to examine their differential effects (Bazhenov et al., 2002), while another model, with either variable changed in isolation, showed that the absence of activity-dependent K^+^ conductances virtually abolished transitions to the Down state, while absence of synaptic depression only diminished the synchrony of these transitions, suggesting a paramount role for K^+^ conductances (Hill and Tononi, 2005).

Proposed mechanisms for Up state termination must explain the high level of synchrony of this event across cortical neurons. The transition to the Down state may be the most synchronous event during the slow oscillation (Volgushev et al., 2006; Mochol et al., 2015). It might seem that a terminating mechanism such as the activation of intrinsic hyperpolarizing currents or the depression of synaptic inputs is incompatible with such a high level of synchrony; intrinsic currents and synaptic depression vary in expression and magnitude from cell to cell and from synapse to synapse. However, given that Up states also *initiate* with a high level of synchrony due to the preferential activation of a certain number of highly excitable or well connected cells (see “Up state initiation” Section), it is also conceivable that Up states can *terminate* synchronously due to decreased excitability in these same cells; the failure of activity in the rest of the network would rapidly follow the decreased excitability of this critical sub-network. The prediction of this account of Up state termination synchrony is that not only the initiation of the Up state but also the termination of the Up state should be a spatiotemporally stereotypical event. That is, activation of a given sub-network should lead to the rapid recruitment of the rest of the network into an Up state, while decreased excitability in this same sub-network should lead to the rapid failure of wider network activity, leading to the Down state. In contrast to Up state onset, however, there is little evidence that Down state onset exhibits a stereotypical neuronal sequence (Volgushev et al., 2006; Luczak et al., 2007). Thus, it is unclear if the termination of the Up state is due to the decreased excitability of the same sub-network responsible for initiating the Up state. Furthermore, such a mechanism for Up state termination does not account for the higher degree of synchrony compared to initiation, unless activity in the critical sub-network is somehow halted more synchronously than it began. Such a scenario is conceivable if, for instance, all of the neurons in this sub-network express an activity-dependent K^+^ conductance to a similar degree, thereby extinguishing activity with smaller temporal lags than are associated with the various synaptic delays within the sub-network. It is important to note that, while the transition to the Down state is a highly synchronous event, the degree of synchrony likely varies greatly with neuromodulatory tone during natural slow-wave sleep and with anesthesia in experimental preparations (Chauvette et al., 2011). Indeed, in the limit, highly local Down states emerging from the “activated” cortical network during wakefulness have been reported (Vyazovskiy et al., 2011). These local Down states, which can be selective enough to involve particular groups of cells within a cortical area, increase in frequency, duration, and spatial extent with time awake. While the mechanisms of these local Down states are unknown, their locality is likely due to the high neuromodulatory tone associated with waking.

Early investigations of the cellular basis of the slow oscillation suggested that the transition to the Down state was likely not precipitated by synaptic inhibition because hardly any cells discharged preferentially in relation to this transition, including putative GABAergic aspiny cells (Contreras and Steriade, 1995), and input resistance monotonically increased during the Up state, suggesting a withdrawal of both excitatory and inhibitory inputs (Contreras et al., 1996b). Estimates of excitatory and inhibitory conductances and their temporal profile during Up states *in vivo* (Haider et al., 2006; Rudolph et al., 2007) and *in vitro* (Shu et al., 2003b; Neske et al., 2015) also support the view that both types of synaptic inputs decrease during the Up state. Thus, the lack of an observed increase in inhibitory cell firing relative to Up state termination and the fact that inhibitory conductances decrease as the Up state progresses has suggested that synaptic inhibition is not an important factor for Up state termination. In fact, progressive pharmacological blockade of fast, GABA_A_-mediated inhibition actually results in a continuous *decrease* in Up state duration *in vitro* (Mann et al., 2009; Sanchez-Vives et al., 2010), perhaps attributable to the enhanced activation of activity-dependent K^+^ conductances due to increased pyramidal cell firing in the disinhibited network (Sanchez-Vives et al., 2010; Igelström, 2013). At the same time, however, slow GABA_B_-mediated inhibition appears to play some role in terminating Up states, since progressive pharmacological blockade of this inhibition results in a continuous *increase* in Up state duration *in vitro* (Mann et al., 2009), an effect that depends on presynaptic GABA_B_ receptors (Craig et al., 2013). Thus, while the firing of inhibitory cells does not seem to occur preferentially in relation to Up state termination, a slower, non-phasic inhibition, due to both the slower kinetics of GABA_B_ receptors and perhaps also the more prolonged GABAergic signaling associated with the extrasynaptic localization of these receptors (Kulik et al., 2003), may play a role. This slow GABAergic process may supplement the similarly slow intrinsic inhibitory process associated with activity-dependent K^+^ conductances.

While, at the population level, inhibitory cells do not increase their firing rate as the Up state progresses and inhibitory synaptic conductance accordingly does not increase, it is still possible that some fraction of inhibitory cells discharge preferentially toward the end of Up states. Consequently, a small, but perhaps significant fraction of excitatory cells would experience an increase in inhibitory synaptic input in the latter portions of an Up state. If this fraction of “late-firing” inhibitory cells has a sufficient degree of axonal spread, then it is possible that synaptic inhibition, while not necessarily apparent at the level of population data, contributes significantly to Up state termination and its characteristic synchrony. One possible candidate for such a “late-firing” inhibitory cell is the somatostatin (SOM)-positive, Martinotti cell, which receives strongly facilitating excitatory synaptic input (Reyes et al., 1998; Gibson et al., 1999; Beierlein et al., 2003; Silberberg and Markram, 2007). In paired recordings of connected SOM-positive inhibitory cells and pyramidal cells during Up states *in vitro*, the spike-triggered average membrane potential of the SOM-positive inhibitory cell was greatest during the last third of the Up state (Fanselow and Connors, 2010), suggesting that this particular inhibitory cell may be most excited near the point of Up state termination. Two cortical network models incorporating facilitating excitatory synapses onto SOM-positive inhibitory cells have suggested that the late firing of these cells during simulated slow oscillations is decisive in terminating Up states (Melamed et al., 2008; Krishnamurthy et al., 2012). The relatively low firing rates (<20 Hz) of individual pyramidal cells during Up states, however, are unlikely to permit effective excitatory facilitation onto postsynaptic SOM-positive inhibitory cells. Indeed, when the temporal profile of firing of these cells during Up states has been characterized, the result has been either a decrease in firing with progression of the Up state (Neske et al., 2015) or a relatively constant level of firing (Fanselow and Connors, 2010). One study has reported a class of fast-spiking inhibitory cells in rat prefrontal cortex that discharges preferentially near the termination of the Up state (Puig et al., 2008), though the source (cortical vs. subcortical) and synaptic basis (excitation vs. disinhibition) for the increased firing of this particular cell during the late portions of the Up state is unclear, as is the generalizability of this firing pattern across cortical areas and species.

Recent evidence from intracellular recordings in anesthetized and sleeping cats combined with computational network models suggest that, while not necessarily apparent in the population firing rate of particular inhibitory cells or the population inhibitory conductance of excitatory cells, synchronized inhibition in a small number of excitatory cells may occur prior to Up state termination. Impelled by their observation that Up state termination is often more synchronized among cortical neurons than initiation (Volgushev et al., 2006), Igor Timofeev and colleagues have reconsidered the possibility that synaptic inhibition increases in certain cortical neurons at the end of Up states. In a computational model of the cortico-thalamocortical network, they showed that either enhancing the intrinsic excitability of cortical inhibitory cells or increasing the strength of excitatory-to-inhibitory cell synapses improved the synchrony of Up state terminations and shortened Up state durations (Chen et al., 2012). Conversely, when the strength of excitatory-to-inhibitory cell synapses was decreased, the duration of Up states increased until reaching a point at which inhibition was so meager that intense excitatory cell firing activated activity-dependent K^+^ conductances, which abruptly terminated network activity. These simulations suggested that synaptic inhibition is critical for the synchronous termination of Up states, while activity-dependent K^+^ conductances might play a more important role in a disinhibited network.

Contrary to the modeling results in Chen et al. (2012), progressive pharmacological blockade of fast synaptic inhibition during Up states *in vitro* progressively *decreased* Up state duration (Mann et al., 2009; Sanchez-Vives et al., 2010; see also above). While the reasons for these discrepancies are unclear, they might be attributed to the differential degree of afferentation of the systems queried in these studies. In Chen et al. (2012), the model includes both the local cortical circuit as well as cortico-thalamocortical loops encompassing both core and matrix thalamic nuclei. In the *in vitro* studies of Mann et al. (2009) and Sanchez-Vives et al. (2010), these loops are not present. It is possible that thalamocortical projections in the model from Chen et al. (2012) have a synchronizing influence on cortical inhibitory cells beyond what the local cortical circuit is capable of achieving (see also “Contribution of the thalamus” Section). It would be interesting to dissect the precise contribution of the cortico-thalamocortical loop to the synchronizing effect of synaptic inhibition on Up state termination by systematically removing components of this loop and examining possible changes in the relative contributions of synaptic inhibition vs. activity-dependent K^+^ conductances in termination.

**Figure 4:**
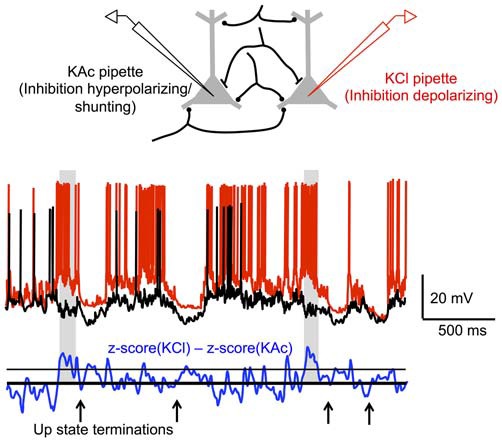
**Increased inhibition during Up state terminations.** (Top) Synaptic inputs are assumed to be predominately shared between nearby (<500 μm) cortical neurons. In recordings with a KAc pipette, inhibition should be hyperpolarizing or shunting, while in recordings with a KCl pipette, inhibition should be depolarizing. Thus, differences between the membrane potential excursions in KAc-KCl dual recordings should predominately reflect inhibition. (Bottom) Dual KAc-KCl recording in sleeping cat cortex. Periods of inhibition (shaded regions) occur above a pre-determined threshold primarily before Up state termination. Adapted by permission from the American Physiological Society: *Journal of Neurophysiology* (Lemieux et al., 2015), © 2015.

Most recently, Timofeev and colleagues have studied the possible contribution of synaptic inhibition to Up state termination with intracellular recordings in anesthetized or sleeping cats. Specifically, by performing dual recordings of nearby (<500 μm) neurons, with one pipette filled with potassium acetate (KAc) and the other filled with KCl, the authors quantified the contribution of synaptic inhibition to the membrane potential during different periods of the Up state (Lemieux et al., 2015). GABA_A_-mediated responses in the cell recorded with KCl would be depolarizing, whereas these same responses would be hyperpolarizing or shunting in the cell recorded with KAc. Since cells separated by <500 μm receive many common synaptic inputs, subtracting the membrane potential excursions in a cell recorded with KAc from those of a nearby cell simultaneously recorded with KCl will primarily reflect GABA_A_-mediated responses. Lemieux et al. (2015) found that when the differences in membrane potential excursions between KAc- and KCl-recorded cells exceeded a particular threshold, they mostly did so during the last few hundred milliseconds prior to Up state termination (Figure 4). These periods of “long-duration inhibition” prior to Up state termination occurred above chance level in 35% of paired recordings and during 10–19% of Up state terminations in these recordings. Thus, at the population level, this implies that synaptic inhibition dominates 4–7% of cortical neurons prior to Up state termination. Whether this elevated synaptic inhibition in a small proportion of cells is decisive in terminating Up states is still unclear. Furthermore, it is still unknown whether this inhibition reflects the synchronized firing of a particular group of inhibitory cells near the end of the Up state, as in Puig et al. (2008). If so, this implies that a very small and selective proportion of inhibitory cells increases their firing rate during the Up state, with the cellular or network mechanisms for such an increase to be determined.

Taking the various *in vivo*, *in vitro*, and *in silico* results discussed above into account, the principal mechanism by which the cortical network transitions from activity to silence during the slow oscillation is likely the activation of activity-dependent K^+^ conductances. A modulatory influence on Up state duration may also be provided by the activation of GABA_B_ receptors due to the extrasynaptic accumulation of GABA resulting from the high activity of inhibitory cells during the Up state. While fast, GABA_A_-mediated inhibitory signaling does not appear to occur preferentially near the end of the Up state at the population level, a role for this type of inhibition in a small group of neurons cannot be discounted. Whether fast synaptic inhibition in a small population of neurons is critical for the termination of the Up state and its characteristic synchronization, or is ancillary to slower inhibitory mechanisms, remains to be determined. Unequivocal evidence for such a prime role for fast synaptic inhibition in a small population of cells would require the identification of these cells, or more desirably the population of presynaptic inhibitory cells whose firing rate presumably increase toward the end of the Up state, and the experimental manipulation of their activity. This is a challenging task, but may be possible at least in *in vitro* preparations with some of the newest techniques for cell-specific optical control of neuronal activity (Packer et al., 2013).

## Contribution of the Thalamus

Since its discovery, the slow oscillation has generally been considered a predominantly cortical phenomenon. The sufficiency of the cortex for the generation of the slow oscillation was suggested based on its survival following thalamic lesions (Steriade et al., 1993b). While expressed at a lower frequency, the slow oscillation can also be recorded from cortical slabs *in vivo* (Timofeev et al., 2000) and cortical slices maintained *in vitro* (Sanchez-Vives and McCormick, 2000; see also “The slow oscillation *in vitro* Section). Thus, the tendency to oscillate at <1 Hz appears to be a fundamental feature of even quite local cortical circuits. It is possible, however, that these observations have led to an underestimation of the role of subcortical structures in shaping the slow oscillation, in particular the thalamus and brainstem neuromodulatory nuclei. Earlier work on the slow oscillation had suggested at least a modulatory role for these subcortical structures, and recent evidence has suggested an even more active role.

The role of the thalamus in regulating the properties of the slow oscillation has been increasingly acknowledged in the ensuing two decades since the original work of Steriade and colleagues. To understand the role of the thalamus during the slow oscillation, it is first important to consider the firing properties of excitatory thalamic relay cells and inhibitory cells of the thalamic reticular nucleus (TRN) during this activity. Steriade and colleagues provided intracellular recordings from these cells in their initial studies, primarily from the ventral lateral nucleus of the thalamus (VL) and the reciprocally connected rostral lateral sector of the TRN (Steriade et al., 1993c; Contreras and Steriade, 1995; Timofeev and Steriade, 1996). Following the Down state, thalamocortical neurons fire a post-inhibitory rebound spike-burst, which is followed by an Up state in the cortex. Corticothalamic input depolarizes TRN cells, causing them to discharge in bursts or tonically at high frequencies. Due to the high level of spiking activity in the TRN, thalamocortical neurons, after their initial spike burst, are quickly overcome by massive synaptic inhibition from the TRN, mostly preventing spiking output. Thus, if thalamocortical projections play a role in the slow oscillation, it would seem that this role is primarily the initiation of Up states and consequently, the determination of the oscillation period. The phasic burst-firing behavior of thalamocortical neurons at the onset of the Up state, which often preceded the firing of cortical neurons by a few tens of milliseconds, indeed prompted Steriade and colleagues to propose that the activity of thalamocortical neurons might provide the trigger for Up states on each cycle of the slow oscillation (Contreras and Steriade, 1995) and contribute to their synchronization across the cortex (Amzica and Steriade, 1995a).

The potential for thalamocortical cells to initiate Up states rhythmically during the slow oscillation can be appreciated when considering the powerful effect of sensory stimulation or direct thalamic stimulation in evoking Up states. In anesthetized animals, both temporally prolonged sensory stimuli, such as drifting gratings (Anderson et al., 2000; Jia et al., 2010) and punctate stimuli, such as brief whisker deflections in rodents (Petersen et al., 2003; Hasenstaub et al., 2007) are effective initiators of Up states in the respective sensory cortices. In slice preparations that preserve axons of thalamocortical neurons (Agmon and Connors, 1991; Cruikshank et al., 2002), electrical or optogenetic stimulation of the thalamus is also capable of triggering Up states (Metherate and Cruikshank, 1999; MacLean et al., 2005; Rigas and Castro-Alamancos, 2007; Watson et al., 2008; Wester and Contreras, 2012; Favero and Castro-Alamancos, 2013; Wester and Contreras, 2013). A thalamic mechanism for Up state initiation could also be manifested during the slow oscillation.

The burst-firing of thalamocortical neurons prior to the discharge of cortical neurons during the slow oscillation is suggestive of a thalamic contribution to Up state initiation, yet this possibility has to a large extent been ignored due to the endurance of the oscillation following thalamic lesions, as well as its presence in cortical slabs and *in vitro* slice preparations. Recent work, however, has revealed a fundamental role for the thalamus in the full expression of the slow oscillation. In mouse barrel cortex *in vitro*, severing thalamocortical axons significantly decreased the frequency of the slow oscillation, indicating that spontaneous thalamic activity in this preparation contributes to the initiation of spontaneous Up states (Rigas and Castro-Alamancos, 2007). Recent *in vivo* studies have also demonstrated an important contribution of thalamic activity to the pacing of the slow oscillation. Acute pharmacological blockade of action potentials in thalamic neurons in anesthetized and naturally sleeping rats decreased the frequency of the slow oscillation (David et al., 2013). Interestingly, selective blockade of T-type Ca^2+^ channels also significantly reduced slow oscillation frequency (David et al., 2013), consistent with the idea that the post-inhibitory rebound spike burst in thalamocortical neurons is an initiation signal for Up states.

**Figure 5:**
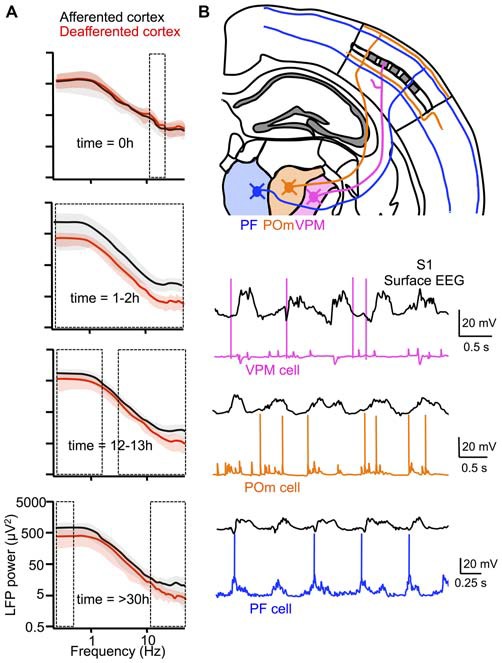
**Active thalamic contribution to the cortical slow oscillation. (A)** Pharmacological inactivation of thalamic nucleus LP in anesthetized cats resulted in deafferentation of certain regions of suprasylvian gyrus (red), but not others (black). Effects of deafferentation on the slow oscillation were quantified by differences in LFP power. Boxed regions indicate statistically significant (*p* <0.05) frequency bins. The slow oscillation and faster oscillatory activities during Up states were significantly affected within ~12 h after thalamic inactivation, but many aspects of the slow oscillation recovered after 1 day, perhaps due to up-regulation of intracortical synaptic connections. **(B)** Distinct activities of core (VPM) and matrix (POm and PF) thalamic nuclei during the slow oscillation in anesthetized mouse. VPM cells are strongly inhibited during the slow oscillation, while POm and PF cells are depolarized in phase with the slow oscillation. Thalamic neurons in matrix nuclei may play an active role in the cortical slow oscillation. **(A)** adapted by permission from the Society for Neuroscience: *Journal of Neuroscience* (Lemieux et al., 2014), © 2014. **(B)** adapted by permission from the Society for Neuroscience: *Journal of Neuroscience* (Sheroziya and Timofeev, 2014), © 2014.

In a seemingly marked deviation from the original work of Steriade and colleagues, Timofeev and colleagues, using the same *in vivo* preparation (i.e., the anesthetized cat), recently showed that thalamic inactivation reduced both the slow oscillation frequency and the occurrence of fast oscillations during Up states (see “Beta/gamma oscillations” Section), and furthermore diminished the synchronization of Up states in simultaneously recorded cortical neurons (Lemieux et al., 2014). In some recordings, slow oscillations were virtually abolished following thalamic lesions. This is in striking contrast to the results of Steriade et al. (1993b) who reported that thalamectomy does not affect the properties of the slow oscillation. The difference between the results of this study and those of the earlier work by Steriade and colleagues is likely attributable to the time after thalamic inactivation that slow oscillations were recorded. In Steriade et al. (1993b), recordings of the slow oscillation were performed 2 days following experimental lesions of the thalamus, whereas in Lemieux et al. (2014), recordings were performed continuously before and after thalamic inactivation. These continuous recordings revealed that while the slow oscillation frequency remained significantly lower up to 12 h following thalamic inactivation, the cortex attained a slow oscillation frequency comparable to its afferented state within 30 h (Figure 5A). This recovery was also evident in a cortical slab preparation. The authors attributed the recovery of the normal slow oscillation frequency to compensatory changes in excitatory synaptic connections, based on the increase of spontaneous EPSPs recorded during the Down state and the recovery of a normal slow oscillation in a “deafferented” thalamocortical network model following the up-scaling of excitatory synaptic connections. Thus, the results from Lemieux et al. (2014) not only demonstrate a major contribution of the thalamus to the full expression of the slow oscillation, but also suggest that this oscillation is of particular importance to cortical networks, since active modifications of local recurrent circuitry appear to compensate for the absence of a thalamic signal for Up state initiation (see also “Functions of the slow oscillation” Section).

Recordings from various thalamic nuclei reveal a possible role for thalamocortical neurons beyond that of a cue for the beginning of each slow oscillation cycle. In particular, whereas inhibition strongly dominates the Up state in relay cells from sensory thalamic nuclei [e.g., ventral posterior medial nucleus (VPM) and lateral geniculate nucleus (LGN)], thus largely preventing spiking at all times expect the very onset of the Up state, excitation dominates in non-sensory thalamic nuclei [e.g., posterior nucleus (PO) and intralaminar nuclei] allowing constituent neurons to spike throughout the duration of the Up state (Sheroziya and Timofeev, 2014; Figure 5B). The basis for this dichotomy in firing pattern probably lies in the intense inhibition of sensory thalamic nuclei by the TRN and the lack of this inhibition in non-sensory thalamic nuclei, which receive the majority of their inhibitory input not from the TRN, but from the zona incerta (Barthó et al., 2002). Notably, while TRN neurons with projections to sensory thalamic nuclei are highly active during the slow oscillation, TRN neurons with projections to limbic thalamic nuclei exhibit low levels of activity (Halassa et al., 2014). Given the preponderance of excitation in non-sensory thalamic nuclei, thalamocortical neurons in these regions are in a position to play an active role not only in Up state initiation, but also in its persistence and perhaps termination. Moreover, a feature of the projections of thalamocortical neurons from many non-sensory thalamic nuclei is a diffuse targeting of multiple cortical territories, in contrast to sensory thalamic nuclei, whose projections are more focal. These distinct projection patterns are often denoted as “matrix” or “core,” respectively (Jones, 1998). Taking into account both the prolonged excitation of thalamocortical neurons of non-sensory thalamic nuclei during Up states and their diffuse projections to cortex, these neurons might not only play an active role during multiple phases of the slow oscillation, but might also be key to the synchronization of the slow oscillation across the cortex. This might explain why in Lemieux et al. (2014) not only did thalamic inactivation impair the slow oscillation, but also the higher-frequency oscillations occurring during Up states; the absence of thalamocortical synaptic activity might also have impaired the synchronization of local cortical network activity.

Thalamic neurons are also endowed with intrinsic ionic currents whose interactions can give rise to slow (<1 Hz), rhythmic oscillations, perhaps serving as at least a partial pacemaker for the cortical slow oscillation. While thalamic neurons *in vitro* generally do not exhibit any spontaneous rhythmic membrane potential fluctuations or spiking, activation of metabotropic glutamate receptors (mGluRs; e.g., through bath-application of appropriate agonists or stimulation of corticothalamic axons) causes a rhythmic slow oscillation in these neurons that is insensitive to blockade of synaptic transmission and spiking activity (Hughes et al., 2002). The mechanism for this cell-intrinsic slow oscillation in thalamic neurons is an mGluR-mediated reduction in the outward current associated with the leak conductance (g_Leak_; McCormick and von Krosigk, 1992), which leads to membrane potential bistability due to the interaction of this current with the inward current associated with the non-inactivating portion of T-type Ca^2+^ channel conductance (i.e., T_Window_). That is, when g_Leak_ is low, there are two stable membrane potentials where the outward current mediated by g_Leak_ is balanced by the inward current mediated by T_Window_, one hyperpolarized and one depolarized (Williams et al., 1997; Hughes et al., 1999). If these were the only conductances activated in these voltage ranges, the membrane potential of thalamic neurons would not oscillate, but rather remain at either of the stable levels until external stimulation toggled between them. This is not the case, however, because the hyperpolarization-activated current (h-current) is also expressed in these neurons, which depolarizes them to the level at which they generate a low-threshold Ca^2+^ spike (mediated by T-type Ca^2+^ channels) and subsequently, due to the stable interaction between the leak current and the current mediated by T_Window_, the membrane potential remains depolarized. The Ca^2+^-dependent nonspecific cation current (I_CAN_) also plays a role in maintaining the depolarized membrane potential of thalamic neurons (Hughes et al., 2002). This depolarized membrane potential is not sustained indefinitely, however, because the h-current inactivates and I_CAN_ decreases, repolarizing the cell to the other (hyperpolarized) stable membrane potential, before the cycle recommences. The same intrinsic slow oscillation is also expressed in TRN neurons (Blethyn et al., 2006). The importance of the h-current for exhibiting this slow oscillation is apparent when it is blocked pharmacologically; short current pulses can then switch thalamic neurons between two stable membrane potential values, where they remain until the another current pulse is applied (Williams et al., 1997; Hughes et al., 1999).

The recent *in vivo* studies demonstrating a dramatic effect of thalamic inactivation on the rhythmicity and synchrony of the cortical slow oscillation, in addition to the observation of an autonomous slow oscillation in thalamic and TRN neurons *in vitro*, has challenged the cortico-centric view of its generation. A complete understanding of the mechanisms of the slow oscillation requires a consideration of the synaptic interactions among all of these components (Crunelli and Hughes, 2010; Crunelli et al., 2015). The potential to be enforced by multiple sites entails the robustness of the slow oscillation, since any deficiencies (e.g., deviations from rhythmicity or synchrony) are likely to be quickly amended due to the redundant expression of the oscillation. This redundancy might be an indication of the importance of the slow oscillation for normal brain function.

## Contribution of the Basal forebrain and Brainstem Nuclei

Other subcortical structures that may actively contribute to the slow oscillation include various brainstem nuclei sending cholinergic, noradrenergic, or serotonergic axons to cortex and thalamus. Cortically projecting cholinergic neurons in the basal forebrain are also in a position to modulate the slow oscillation. While the slow-wave sleep state in which the slow oscillation occurs is associated with generally low neuromodulatory tone, neurons in these subcortical nuclei are not completely silent. Neurons firing with distinct preference for either the Up or Down state have been found in the basal forebrain (Détári et al., 1997; Manns et al., 2000), pedunculopontine tegmentum (PPT; Mena-Segovia et al., 2008), locus coeruleus (LC; Eschenko et al., 2012), and dorsal raphe nucleus (DRN; Schweimer et al., 2011). Whether the phasic or tonic firing properties of neurons in these various subcortical areas are a signature of an active role in shaping the slow oscillation or merely a transfer of activity from presynaptic cortical neurons that project to these areas still must be resolved.

The causal role of brainstem nuclei as “activating systems” (i.e., extinguishers of slow-wave EEG patterns) has been well-documented since the pioneering work of Moruzzi and Magoun (1949). A causal role for brainstem nuclei in the control of slow waves themselves, however, has been less studied, likely due to the assumption that the contribution of these nuclei to cortical or thalamic activity is negligible except in states of cortical activation. Additionally, in slice preparations, application of agonists for neuromodulator receptors often abolishes spontaneous and evoked Up states (Hsieh et al., 2000; Favero et al., 2012; Wester and Contreras, 2013), likely due to the suppression of release probability in intracortical synapses (Gil et al., 1997). Nevertheless, a functional, while small, level of neuromodulatory tone may exist in cortical and thalamic networks during the slow oscillation. One of the first indications that neuromodulatory signals arising from subcortical structures could be important for the slow oscillation came from Steriade and colleagues shortly after their initial characterization of the oscillation. They determined that while electrical stimulation of the PPT and LC caused “activation” of the cortical EEG (i.e., abolition of slow waves; see “Up state termination” Section), blockade of muscarinic cholinergic signaling during the baseline, slow-wave state reduced the duration of Up and Down states (i.e., shorter Up states occurring at higher frequency; Steriade et al., 1993d). A recent *in vitro* study of mouse sensory and frontal cortex has proposed a role for cholinergic afferents specifically in maintaining the rhythmicity of the slow oscillation (Lörincz et al., 2015). An *in vivo* component of this study also replicated the result of Steriade et al. (1993d) that the slow oscillation depends on intact cholinergic tone. Similar to other neuromodulators, the cellular and synaptic effects of acetylcholine are quite complex, being dependent on receptor subtype, pre- vs. postsynaptic expression of receptor, neurotransmitter concentration, and differential expression of receptors in different excitatory and inhibitory neuron subtypes (McCormick, 1992; Muñoz and Rudy, 2014). Thus, it will be challenging to decipher the precise mechanisms of cholinergic modulation of the slow oscillation.

Serotonergic signaling has a long and controversial history in relation to the slow oscillation and sleep. In the 1960’s, studies employing lesions of the DRN and chemical inhibition of serotonin synthesis resulted in strong and long-lasting insomnia in cats, leading to the hypothesis that serotonin was a crucial, if not the sufficient neuromodulator for initiating slow-wave sleep (Jouvet, 1972, 1999). Subsequent recordings from the DRN during the sleep-wake cycle, however, showed that neurons substantially decreased their firing from behavioral arousal to slow-wave sleep (McGinty and Harper, 1976; Trulson and Jacobs, 1979), a conspicuous inconsistency with the serotonergic hypothesis of sleep. While probably not the nexus of sleep promotion it was once proposed to be, the serotonergic system of the DRN may play a role in the slow oscillation in several ways. First, while DRN neurons as a population considerably decrease their firing from waking to sleep, a heterogeneity of firing patterns exists among different types of DRN neurons, with 12% of cat DRN neurons actually exhibiting an increase in firing rate during slow-wave sleep compared to waking (Sakai and Crochet, 2001). In anesthetized rats, most serotonergic DRN neurons fired phasically with the cortical slow oscillation, though interestingly with a preference for the Down state (Schweimer et al., 2011). Thus, at least a fraction of DRN neurons can influence their synaptic targets during the slow oscillation. Second, serotonin differentially modulates the excitability of thalamocortical and TRN neurons, in the same direction as these cells are affected during the slow oscillation. That is, while serotonin depolarizes TRN neurons (McCormick and Wang, 1991), it hyperpolarizes thalamocortical neurons (Monckton and McCormick, 2002). Although the pronounced excitation in TRN neurons and pronounced inhibition in thalamocortical neurons during the slow oscillation is no doubt predominately due to the stronger unitary excitation of TRN neurons than of thalamocortical neurons by corticothalamic afferents (Golshani et al., 2001), serotonergic tone in the thalamus may augment the divergent excitability of TRN and thalamocortical neurons during the slow oscillation. Lastly, the high activity of DRN neurons during waking may *indirectly* affect the properties of the slow oscillation through a homeostatic up-regulation of sleep-promoting neurotransmitters. According to the homeostatic hypothesis of sleep regulation, the length of time spent in the waking period is proportional to the length of subsequent slow-wave sleep due to the exponential, but saturating increase in some homeostatic process during waking that decreases exponentially during sleep (Borbély, 1980). There is evidence that the serotonergic system engaged during waking plays a role in such a homeostatic process. For instance, chemical inhibition of serotonin synthesis throughout, but not at the end of sleep deprivation, abolishes slow-wave sleep, suggesting that the onset of sleep requires the serotonin-dependent accumulation of hypnogenic factors during waking (Sallanon et al., 1983). Thus, while mostly inactive during the slow oscillation, DRN neurons may contribute to the properties of the oscillation by virtue of the biochemical changes they bring about through high levels of activity during waking.

## Functions of the Slow Oscillation

On first consideration, it is curious that the slow oscillation, a recurrence of robust and synchronized activity, is associated with deep sleep, a condition in which the cortex is largely disconnected from the sensory environment. Why is a behavioral state bereft of cognitive or sensory processing associated with such rich spontaneous activity? Indeed, the neuronal activity during the slow oscillation that has been detailed in cortical and thalamic networks over the past two decades would undoubtedly have surprised early psychologists and neurophysiologists who viewed sleep as a manifestation of behavioral inhibition (Pavlov, 1923) or “abject mental annihilation” (Eccles, 1961). While the network-wide cycling between activity and quiescence during the slow oscillation is highly suggestive of a functional role, it could be argued that the salience of this phenomenon alone is not sufficient to afford it functionality. It could be that Up and Down states are simply default activity patterns that unavoidably follow from the recurrent network architecture of the cortex. As much is suggested by the fact that this activity occurs even in cortical slices *in vitro* under conditions of slightly enhanced excitability. Nevertheless, even if the slow oscillation primarily owes itself to the basic architecture of the cortex, a functional role for the slow oscillation may still exist.

When slow-wave sleep is viewed not as a mere idling of the brain, but as an active period in its own right, associated with the functional reorganization of neuronal networks across wide cortical territories, the possible functions of the slow oscillation become more clear. These possible functions include the synchronization of higher-frequency oscillations, the consolidation of memory traces acquired during waking, and basic biochemical maintenance in neurons during Down states.

### Grouping of Higher-Frequency Oscillations by the Slow Oscillation

The cerebral cortex generates a panoply of oscillations distinguished by their different frequency bands (Buzsáki, 2006). Except in artificial circumstances, cortical oscillations of one frequency band are rarely expressed alone. Rather, oscillations of different frequencies interact in various ways through relations between their amplitudes and phases. For instance, one of the earliest demonstrations of the interactions between two different oscillations was the modulation of the amplitude of gamma (30–80 Hz)-band activity by the phase of slower theta (4–10 Hz)-band activity in the rat hippocampus (Buzsáki et al., 1983). Such phase-amplitude coupling could provide a mechanism for integrating the results of neural computation across multiple spatial and temporal scales (Canolty and Knight, 2010) and allow for information to be processed in a hierarchical manner (Buzsáki, 2010). During slow-wave sleep, the slow oscillation serves as a “base frequency” that groups the two other major oscillations associated with slow-wave sleep, spindle and delta oscillations. The Up states of the slow oscillation are furthermore often associated with an increase in beta/gamma-band activity, frequencies that are often considered hallmarks of the waking, information-processing state.

#### Spindle Oscillations

Spindle oscillations (7–14 Hz) are one of the distinctive features of slow-wave sleep. Rhythmogenesis of spindles is due to the discharge of spike-bursts by GABAergic neurons of the TRN, which impart rhythmic IPSPs onto thalamocortical neurons. At sufficiently hyperpolarized potentials, as is the case during slow-wave sleep, thalamocortical neurons can fire rebound spike-bursts after each IPSP, imparting spindle-frequency excitatory input to cortex. In many respects, the TRN is a pacemaker for spindles, as evidenced by, for instance, the persistence of spindles when the TRN is separated from the thalamus (Steriade et al., 1987) and the sufficiency of optogenetic excitation of TRN cells to generate spindles (Halassa et al., 2011).

While the TRN may be sufficient for the rhythmicity of spindles, it is not sufficient for their characteristic synchrony across the thalamus and cortex. The synchronous occurrence of spindles requires active corticothalamic input impinging on TRN cells, since the removal of cortex results in a temporal disorganization of spindles across thalamic recording sites (Contreras et al., 1996a). Furthermore, spindles occurring *in vitro* do not occur synchronously, but propagate as a traveling wave (Kim et al., 1995), likely due to the absence of the synchronizing action of corticothalamic inputs. The origin of the synchronizing corticothalamic activity is the spiking of cortical cells during the Up state. Thus, when they occur synchronously (to the extent that they can be recorded with EEG), spindles quickly follow the initial depolarization of the Up state in cortical neurons (Steriade et al., 1993c), a sequence known as a “K-complex” in the clinical EEG literature (Amzica and Steriade, 1997). Thus, there is a consistent phase relationship between Up states and spindles.

#### Delta Oscillations

Delta oscillations (1–4 Hz) are most common during the deepest stages of slow-wave sleep. There appear to be at least two rhythmogenic mechanisms for the delta oscillation, one relying solely on the intrinsic properties of thalamocortical neurons, and the other likely relying on the intrinsic properties of layer 5 pyramidal neurons. The ionic currents underlying the delta oscillation arising from thalamocortical neurons have been well-characterized. At membrane potentials more hyperpolarized than those associated with spindles, interactions between the h-current and the T-current (see “Contribution of the thalamus” Section) produce delta rhythmicity: the h-current is activated and the T-current is de-inactivated, which leads to the activation of the T-current and the emergence of a low-threshold calcium spike (often crowned by sodium spikes), which deactivates the h-current and inactivates the T-current, repolarizing the cell such that the h-current can begin the cycle again (McCormick and Pape, 1990). The requisite hyperpolarization of thalamocortical cells for the interaction of the h-current and T-current to give rise to delta oscillations is caused by the even further decrease in firing of brainstem cholinergic neurons during deep slow-wave sleep (Steriade et al., 1991b).

The existence of a separate cortical generator of the delta oscillation was suggested after its persistence following removal of the thalamus (Steriade et al., 1993b). The ionic basis for the cortically generated delta oscillation is not known at the same level of detail as the thalamically generated one, but it may incorporate many of the same mechanisms involved in the alternation of Up and Down states during the slow oscillation (i.e., the activation of intrinsic bursting pyramidal neurons in layer 5 and the subsequent activation of long-lasting K^+^ conductances; see “Up state initiation” and “Up state termination” Section). Indeed, recent observations in quiet-waking rodents call into question the notion that delta oscillations, at least the cortically generated variety, are only associated with slow-wave sleep. Petersen et al. (2003) first reported ~2 Hz oscillations in the membrane potential of cortical neurons in quiet-waking mice and rats. Carl Petersen’s group has subsequently recorded these synchronized membrane potential oscillations in waking mice in several reports (e.g., Crochet and Petersen, 2006; Poulet and Petersen, 2008). Delta-frequency oscillations during waking cannot originate from thalamus because thalamocortical neurons are too depolarized to activate the h-current and de-inactivate the T-current. Instead, these oscillations occurring at traditional delta frequencies during the waking state are probably mechanistically indistinguishable from cortical Up and Down states. Such a high-frequency “slow oscillation” might be attributable to a level of K^+^ conductance as high as that during slow-wave sleep, yet perhaps in concert with cortical activity levels or synaptic transmission properties comparable to those associated with waking. Indeed, cholinergic signaling in cortex increases from quiet wakefulness to active behavior (Eggermann et al., 2014), which would be associated with the closing of K^+^ conductances that perhaps promote delta-frequency membrane potential oscillations.

During deep slow-wave sleep, cortical Up states synchronize thalamically generated delta oscillations in a manner similar to their synchronization of spindles during lighter slow-wave sleep. While thalamocortical neurons can generate delta oscillations autonomously, there is no intrathalamic mechanism to synchronize the oscillation among thalamocortical neurons since these cells primarily lack any recurrent collaterals. The synchronization of the thalamic delta oscillation is attributable to monosynaptic excitatory input from corticothalamic cells that are depolarized during the Up state, as well as disynaptic inhibition from TRN cells (Steriade et al., 1991b). The cortical enforcement of delta-band synchrony among bursting thalamocortical neurons has the same functional consequence as in spindles: a massive postsynaptic effect in the dendrites of cortical neurons, perhaps promoting signaling cascades associated with plasticity (see “Synaptic plasticity and the slow oscillation” Section).

#### Beta/Gamma Oscillations

During states of cortical activation, which include REM sleep and the waking state, slow and globally synchronized oscillations are replaced with faster and more locally synchronized oscillations, such as in the beta (15–30 Hz) and gamma (30–80 Hz) range. While usually associated with sensory processing and higher-level mental activity, synchronized fast oscillations also occur during the Up states of the slow oscillation (Steriade and Amzica, 1996; Steriade et al., 1996a,b). This observation led Steriade and colleagues to suggest that synchronized fast oscillations are not a special quality of the waking, information-processing state, but a general characteristic of depolarization in cortical and thalamic neurons. This notion was based on the ability of cortical and thalamic neurons to exhibit sub- and suprathreshold fast oscillations under appropriate depolarizing current injection (Llinás et al., 1991; Steriade et al., 1991a; Nuñez et al., 1992; Gray and McCormick, 1996). Fast oscillations in cortical networks are also critically dependent on the synchronized firing of fast-spiking inhibitory interneurons, which is a phenomenon associated with Up states of the slow oscillation (Hasenstaub et al., 2005). The suggested functional roles of synchronized fast oscillations in cortical processing during waking are myriad (Fries, 2009; Bosman et al., 2014; Pritchett et al., 2015), though their roles in slow-wave sleep, if any, are uncertain. The fast oscillations associated with REM sleep have been proposed to be a correlate of dreaming mentation (Llinás and Ribary, 1993). While dreams have primarily been considered the province of REM sleep, they also occur during slow-wave sleep, though with different content (Hobson et al., 2000; McNamara et al., 2010). Fast oscillations during Up states of the slow oscillation during slow-wave sleep may provide the framework for dreaming mentation during this stage of sleep.

### Synaptic Plasticity and the Slow Oscillation

The globally synchronized nature of the slow oscillation promotes the synchronization of faster sleep oscillations, particularly those generated in the thalamus, as discussed above. Thus, a major role of the slow oscillation is to modulate other oscillations. Yet, this explanation essentially displaces to another level the original question: is the slow oscillation functional or is it a byproduct of the basic architecture of the cortex? Are thalamic spindle and delta oscillations, potentiated by the cortical slow oscillation, also simply epiphenomena that arise when the brain is disconnected from the sensory environment?

One of the most prominent hypotheses regarding the function of slow-wave sleep, during which the slow oscillation is the cardinal form of neural activity, is that this state allows for the consolidation of memories. While the association of increased synaptic plasticity with sleep was first articulated by Moruzzi (1966) and Steriade and Timofeev (2003), behavioral and physiological experiments testing the dependance of learning and memory on sleep did not begin in earnest until the early 1990’s. At that time, increasing research on a link between sleep and memory consolidation was largely catalyzed by two articles (see comment in Barinaga, 1994): one demonstrating a positive relationship between REM sleep and performance on a learned visual discrimination task in humans (Karni et al., 1994), and another reporting the replay of waking patterns of hippocampal place cell activity during slow-wave sleep in rats (Wilson and McNaughton, 1994). Since the publication of these two articles, there has been a wealth of studies examining relations between sleep and memory in various model organisms and at levels of analysis spanning the molecular to the behavioral (reviewed in Walker and Stickgold, 2004; Waters and Helmchen, 2006; Stickgold, 2005; Massimini et al., 2009; Diekelmann and Born, 2010; Tononi and Cirelli, 2014). Nevertheless, the interpretation of the results of these various studies as conclusive demonstrations of the dependance of learning and memory processes on sleep remains controversial (Vertes, 2004; Frank and Benington, 2006).

As alluded to in “Grouping of higher-frequency oscillations by the slow oscillation” Section, the synchronous burst-firing of thalamocortical neurons during slow-wave sleep, suggests a recruitment of signaling cascades involved in synaptic plasticity in cortical neurons.

Thalamocortical neurons firing synchronous bursts would strongly depolarize the dendrites of postsynaptic pyramidal cells, leading to a massive dendritic Ca^2+^ influx (Yuste and Tank, 1996). Ca^2+^, acting as a second messenger (Ghosh and Greenberg, 1995), can subsequently set into motion signaling cascades associated with increased synaptic plasticity, such as those involving Ca^2+^/calmodulin-dependent kinase II (CaMKII) (Soderling and Derkach, 2000).

A role for the cortical slow oscillation in memory consolidation is also suggested based on its coordination with hippocampal activity during slow-wave sleep. The promotion of synaptic plasticity in the cortex via Ca^2+^-dependent processes coupled with the entrainment of hippocampal activity by cortical Up states may permit the transfer of memory traces from their more labile form in hippocampal networks to their consolidated form in the neocortex. The initial discovery of hippocampal replay of waking patterns of activity during slow-wave sleep (Wilson and McNaughton, 1994) suggested a possible dialogue between hippocampus and neocortex during this period. Since then, there have been many demonstrations of temporal coordination in firing between cortical and hippocampal rhythms during slow wave sleep, with a particular emphasis on the occurrence of sharp-wave ripples (~200 Hz) in hippocampus shortly after the initiation of cortical Up states (Siapas and Wilson, 1998; Sirota et al., 2003; Isomura et al., 2006). Furthermore, hippocampal replay of a sensory experience is coordinated with replay in the associated sensory cortex during slow-wave sleep, with the onset of replay in the cortex apparently driving that in the hippocampus (Ji and Wilson, 2007). Thus, a current working hypothesis for slow-wave-dependent memory consolidation is that cortical Up states, traversing the entorhinal cortex, bias the occurrence of sharp-wave ripples in the hippocampus, which repeatedly transfer hippocampal activity patterns, acquired during learning, to the cortical network in the midst of an Up state, during which the disposition for cortical plasticity is high (Sirota and Buzsáki, 2005).

### The Slow Oscillation as a Period of Cellular Restoration

Down states, periods of synchronously enforced neuronal quiescence, are arguably the most conspicuous feature of the slow oscillation, since network-wide hyperpolarizing periods are rare during waking. While the hypothesis that slow-wave sleep promotes synaptic plasticity and memory consolidation primarily emphasizes the occurrence of Up states, another leading hypothesis of the function of slow-wave sleep focuses on the occurrence of Down states: during these slow-wave-defining periods of network silence, neurons can engage in various restorative and cellular maintenance functions. The notion that slow-wave sleep serves functions besides memory consolidation is conveyed by the various cognitive impairments associated with sleep deprivation (Goel et al., 2009; Killgore, 2010; McCoy and Strecker, 2011; Van Dongen et al., 2011). Indeed, in the most extreme case of sleep deprivation, fatal familial insomnia (Cortelli et al., 1999), slow-wave sleep is impossible, inevitably leading to death. Thus, slow-wave sleep seems to be a period associated with basic repair after the high metabolic demand of the waking state, and perhaps prophylactic measures that counteract further cellular attrition accompanying sustained neuronal activity. Such basic restorative roles during the slow oscillation are likely to be associated with the Down state (Vyazovskiy and Harris, 2013).

## The Relation Between Up States and Activated States

During the Up states of the slow oscillation, cortical neurons exhibit membrane potential dynamics and spiking activity nearly indistinguishable from those properties during the “cortical activation” of REM sleep and waking. In addition, the fast oscillations that typify the activated cortex are also present during Up states (see “Beta/gamma oscillations” Section). Thus, in many respects, Up states resemble abbreviated periods of cortical activation. It can consequently be argued that Up states trigger network operations that are very similar to the essence of waking cortical behavior. From this standpoint, the Up state can be considered a model of the cortex in the active, information-processing state (see “Up states as a model of cortical gain control” Section).

The similarities between the Up states of the slow oscillation during natural sleep or anesthesia and cortical activation during waking span multiple spatiotemporal scales, often as measured in the same preparation. For instance, waking activity and slow-oscillation Up states are characterized by similar relations between multiunit discharges and LFP and by similar spatiotemporal coherence of fast oscillatory activity (Destexhe et al., 1999). The similarity between Up states during slow-wave sleep and waking cortical activity also extends to the relative contribution of excitatory and inhibitory synaptic conductances that drive membrane potential fluctuations: during both waking activity and Up states, the magnitude and variance of inhibitory conductance dominates that of excitatory conductance (Rudolph et al., 2007). One difference between Up states of the slow oscillation and waking activity is the input resistance of cortical neurons. While both types of activity are high-conductance states, in which synaptic activity decreases input resistance by several times the value associated with quiescent periods (i.e., during Down states or *in vitro*; Paré et al., 1998; though, see Waters and Helmchen, 2006), input resistances are higher during waking activity than during Up states (Steriade et al., 2001), perhaps due to the blockade of K^+^ conductances by muscarinic acetylcholine receptors during waking (see “Up state termination” Section).

While the cortex is largely disconnected from the sensory environment when the slow oscillation occurs, the presence of waking-like segments of network activity during this period suggests the cortex is processing internally generated signals, perhaps related to memory consolidation (see “Synaptic plasticity and the slow oscillation” Section) or dreaming mentation (see “Beta/gamma oscillations” Section). Of course, the fragments of cortical activation during Up states do not lead to conscious awareness, so there must be distinguishing factors of veritable waking activity in the cortex that are not reflected during Up states. These factors may include: dynamic variables that cannot be detected with the current spatiotemporal resolution of current electrophysiological or imaging methods, the brevity of cortical activation during Up states (conscious awareness may require longer periods of activation), or diminished long-range effective cortical connectivity during the slow oscillation (Massimini et al., 2005; Destexhe et al., 2007).

## Cortical Responsiveness During Up and Down States

Compared with the Down state, characterized by the almost complete absence of action-potential-dependent synaptic activity in the cortex, the Up state is characterized by vigorous synaptic activity in many cortical neurons. Synaptic activity can have diverse effects on the ability of cortical neurons to respond to afferent inputs. The diversity of the effects of synaptic activity on neuronal responsiveness stems from the various cellular and network properties altered by this activity. The combination of these changes can have complex effects on the ability of cortical neurons to respond to incoming signals. The effects of Up states on neuronal responsiveness have been studied in both *in vitro* and *in vivo* preparations, using various intracellular, sensory, or afferent-pathway stimulation protocols.

### Up States Affect Intrinsic Responsiveness

At the single-neuron level, synaptic activity associated with the Up state causes three primary changes: membrane potential depolarization, increased membrane conductance, and increased membrane potential variance. Each of these changes modulates the neuronal input-output function differently. The neuronal input-output function is defined as the relation between stimulus strength and action potential output. Combining the *in vitro* slow oscillation with the application of dynamic clamp, McCormick et al. (2003) and Shu et al. (2003a) dissected the contribution to the neuronal input-output function of each of the three primary changes associated with Up states. Up states themselves enhance the sensitivity of neurons to synaptic inputs, shifting the input-output function to the left, and selectively enhance the responsiveness to small inputs that are normally subthreshold during the Down state, which is associated with a decreased slope of the input-output function. The leftward shift in the input-output function is primarily due to membrane potential depolarization, which overcomes the rightward shift due to increased membrane conductance. The selective enhancement of responsiveness to small inputs is caused by increased membrane potential noise. An additional effect of the Up state is an overall enhancement of the fidelity of signal transmission: compared to Down states, Up states decrease the latency between the arrival of a synaptic input and the firing of an action potential, enhance spike-timing precision, and improve the cross-correlation between action potential output and complex stimulus waveforms.

### Up States Affect Synaptic Properties of Thalamocortical and Corticothalamic Afferents

The intrinsic membrane properties of cortical neurons constitute one dimension of responsiveness that Up states can modulate. The release properties and synaptic targets of different afferents onto the cortical network are other variables potentially modulable by Up state activity, sometimes in the opposite direction from changes in intrinsic excitability. The effects of Up states on the responsiveness of cortical neurons to stimulation of either thalamocortical or corticocortical afferents has primarily been studied in the somatosensory thalamocortical slice preparation *in vitro* (Agmon and Connors, 1991).

Combining calcium imaging and intracellular recording in thalamocortical slices, MacLean et al. (2005) and Watson et al. (2008) determined that not only did thalamic stimulation elicit Up states that were spatiotemporally indistinguishable from those occurring spontaneously, but also that this stimulation did not perturb spontaneous Up states. These *in vitro* results were consistent with earlier reports in either anesthetized or quiet-waking rodent barrel cortex, in which whisker-stimulation-evoked cortical responses were suppressed by Up states, compared to Down states (Petersen et al., 2003; Sachdev et al., 2004; see also “Up states affect sensory-evoked responses” Section). There are several reasons why Up states might suppress thalamocortical responses: decreased driving force for thalamocortical excitation and increased driving force for thalamocortical feedforward/intracortical inhibition, shunting of thalamocortical PSPs by increased membrane conductance, increased spike threshold, and lower release probability at thalamocortical synapses. Thus, while neurons may be intrinsically more excitable during Up states, the effect of Up states on the response to synaptic input can be suppressive if, for instance, afferent synapses are tonically depressed due to ongoing activity (Reig et al., 2006) or the reversal potential of the thalamocortical synaptic response is close to the level of membrane potential depolarization during the Up state. Another *in vitro* study, however, showed that Up states enhanced cortical responses to thalamic stimulation, but suppressed cortical responses to local cortical stimulation (Rigas and Castro-Alamancos, 2009). The authors of this study attributed the enhanced thalamocortical response primarily to depolarization during the Up state and the suppressed intracortical response to reduced release probability resulting from ongoing activity in the cortex, which is mostly lacking in the thalamus in slice.

### Up States Affect Sensory-Evoked Responses

The results reviewed so far suggest that the interaction between Up states and afferent synaptic input is not straightforward. While depolarization and increased membrane potential variance generally enhance cellular excitability, especially when inputs are small, the ongoing dynamics of the presynaptic afferents that are stimulated and the precise mixture of excitation and inhibition that make up the synaptic response will also determine the direction in which the occurrence of an Up state affects the probability of spiking upon afferent stimulation. If the afferents are already tonically depressed from ongoing activity and if the mixture of excitation and inhibition composing the synaptic response causes the reversal potential of this response to lie near the mean value of Up state depolarization, Up states will suppress neuronal responsiveness compared to Down states. Additionally, while the enhancing effect of depolarization during the Up state has been shown to overcome the accompanying suppressing effect of increased membrane conductance (Shu et al., 2003a), it is conceivable that this relationship might not hold for every Up state or for the entire time course of any given Up state. Diverse effects of Up states on cortical responses to sensory stimuli have been reported *in vivo*, as discussed below.

Early studies investigating the dependance of evoked cortical responses on spontaneous network activity demonstrated a linear relationship between the spontaneous level of the membrane potential in cortical neurons and the magnitude of their evoked response. Such a linear relationship between spontaneous membrane potential and evoked response was described in anesthetized cat motor cortex while stimulating pre-thalamic afferents onto VL (Timofeev et al., 1996) and in anesthetized cat visual cortex while presenting drifting gratings (Arieli et al., 1996; Azouz and Gray, 1999). Later work in anesthetized cat visual cortex also showed a linear relationship between Up-state membrane potential and visually evoked spiking output, with a further demonstration that Up-state depolarization multiplicatively scales the contrast-response function (Haider et al., 2007).

**Figure 6:**
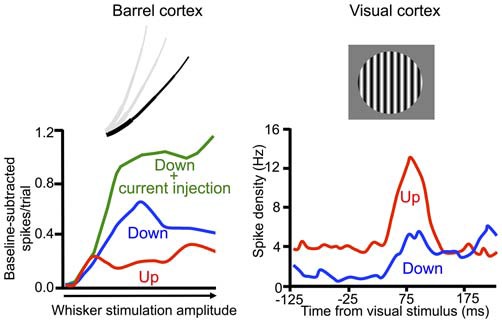
**Up states differentially affect sensory-evoked responses of cortical neurons in barrel and visual cortex.** (*Left*) In rat barrel cortex, whisker stimulation evokes a proportionally larger spiking output during the Down state compared to the Up state. Decreased driving force due to depolarization during the Up state is unlikely to explain this effect since it is not seen with depolarizing current injection during the Down state. Network effects (e.g., thalamocortical synaptic depression or increased intracortical synaptic inhibition) are more likely to account for reduced responsiveness in barrel cortex neurons during the Up state compared to the Down state. (*Right*) Conversely, in cat visual cortex, drifting-grating stimuli evoke larger spiking output during Up states compared to Down states. *Left* adapted by permission from the Society for Neuroscience: *Journal of Neuroscience* (Hasenstaub et al., 2007), © 2007. *Right* adapted by permission from the American Physiological Society: *Journal of Neurophysiology* (Haider et al., 2007), © 2007.

Results from rodent somatosensory (barrel) cortex, however, have generally contradicted those in the visual system. VSD imaging of cortical responses to whisker deflection have revealed lower-amplitude and more spatially confined responses during Up states compared with Down states (Petersen et al., 2003; Civillico and Contreras, 2012). A quenching effect of Up states on whisker-evoked responses in barrel cortex is also evident from the reduced spiking output of single cortical neurons compared to the Down state (Sachdev et al., 2004; Hasenstaub et al., 2007; Figure 6). Discrepancies between visual and somatosensory cortex regarding the influence of Up states in sensory responsiveness could have a number of explanations. As suggested above, the relation between the reversal potential of the sensory-evoked synaptic response and the average membrane potential during the Up state is one factor that could determine the direction in which the Up state affects responsiveness. If the sensory-evoked synaptic reversal potential is comparable to the membrane potential during the Up state, there will hardly be any driving force for the sensory response during the Up state. The relative combinations of excitatory and inhibitory conductances recruited by sensory stimulation determine the sensory-evoked synaptic reversal potential. It could be that the precise combination of these conductances leads to a more hyperpolarized reversal potential in barrel cortex compared to visual cortex. In support of the notion that low driving force during the Up state accounts for the reduced effectiveness of sensory stimulation compared to the Down state, sensory-evoked PSPs in barrel cortex neurons were significantly larger during Down states compared to Up states (Sachdev et al., 2004); in visual cortex, no significant difference was found between PSPs evoked in the Up vs. the Down state (Haider et al., 2007). In contrast, injection of depolarizing current during the Down state in barrel-cortical neurons to maintain the membrane potential at the level encountered during Up states enhanced spiking output to whisker stimulation (Hasenstaub et al., 2007; Figure 6), which is inconsistent with the notion that a reduction of driving force alone accounts for reduced sensory-evoked responses during the Up state. Additionally, consistent with *in vitro* work (Shu et al., 2003a; see “Up states affect intrinsic responsiveness” Section), injection of artificial PSPs into barrel-cortical neurons more readily elicited spiking during Up states than during Down states (Hasenstaub et al., 2007).

Thus, the diminished response in barrel cortex to whisker deflection during Up states does not seem attributable to properties intrinsic to neurons, such as their depolarization, spike threshold, or membrane conductance, but rather to network effects. Network-level mechanisms for decreased responsiveness to whisker stimulation during Up states in barrel cortex likely involve an overall decreased stimulus-evoked synaptic conductance compared to the Down state, most likely due to depression of thalamo- and corticocortical synapses during network activity, and a relative increase of the contribution of inhibition over excitation to the stimulus-evoked synaptic conductance during the Up state compared to the Down state. Both of these possibilities were suggested via estimates of stimulus-evoked synaptic conductance and the contributions of excitation and inhibition to this conductance in Up vs. Down states in barrel cortex (Hasenstaub et al., 2007). One would therefore predict that in visual cortex, where Up states enhance responses to sensory stimuli, stimulus-evoked synaptic conductances are not significantly smaller in the Up state compared to the Down state, or that evoked inhibition is not comparatively larger in the Up state compared to the Down state. Whether there is a difference between barrel cortex and visual cortex in these regards is not known. Interestingly, whereas the response to brief whisker deflections is diminished by the occurrence of Up states, responses to temporally prolonged whisker stimulation are enhanced (Hasenstaub et al., 2007). Thus, the effect of Up states on sensory-evoked responses in cortex can also depend on the dynamics of the stimulus.

Intensity of the sensory stimulus is another possible factor that determines the direction in which Up states modulate sensory responses in cortex. This issue was recently addressed in rat auditory cortex, in which a range of sound stimulus intensities was used, and whose effects on cortical responses were compared between Up and Down states (Reig et al., 2015). In this study, the direction in which Up states modulated neuronal responsiveness during sensory stimulation depended on stimulus strength: responses to weak stimuli were potentiated and responses to strong stimuli were diminished by Up states. The authors attributed the stimulus-intensity-dependent effect of Up states on cortical responses to the competition between thalamocortical network excitability and neuronal membrane conductance, both of which increase during Up states. In essence, high network excitability, associated with Up states, favors low-intensity stimuli, whereas low membrane conductance, associated with Down states, favors high-intensity stimuli. Another possible mechanism for the stimulus-intensity-dependent effect of Up states on neuronal responsiveness (i.e., potentiation at low intensities and suppression at high intensities) was suggested by Shu et al. (2003a), who described an identical effect using artificial EPSPs as stimuli during both natural Up states and simulated Up states via the injection of conductance noise. In this case, noise facilitates spiking probability to small synaptic inputs (those that would cause spiking <50% of the time during the Down state) due to a floor effect (only the depolarizing components of the noise can affect spiking probability), yet diminishes spiking probability to large synaptic inputs (those that would cause spiking >50% of the time during the Down state) due to a ceiling effect (only the hyperpolarizing components of the noise can affect spiking probability). Thus, stimulus-intensity-dependent effects of Up states on neuronal responsiveness could be due solely to the effects of membrane potential variance on individual cortical neurons.

## Up States as a Model of Cortical Gain Control

Since the onset and termination of Up states is quite rapid (a few tens of milliseconds), such periods of enhanced cortical network activity could provide a substrate for the moment-to-moment changes in the routing of information by the cortex, such as is required for processing the context of sensory stimuli, maintaining objects in working memory, or attention. While veritable “Up states,” as distinguished from intervening hyperpolarized periods, have only been unequivocally observed during slow-wave sleep, under certain types of anesthesia, and to some degree to during quiet wakefulness, the rapid increases in synaptic barrages in the cortical network that characterize Up states might also characterize the rapid changes in excitability that would be required for the flexible processing of information (McCormick et al., 2004; McCormick and Yuste, 2006; Haider and McCormick, 2009). In essence, the same neuronal machinery that is spontaneously engaged during sleep may also be adapted by the waking cortex to adaptively route signals on a fast time-scale according to behavioral demands. Consequently, investigation of the cellular and network characteristics of Up states not only provides an understanding of the nature of cortical activity during sleep, but may also provide insight into the mechanisms associated with rapid changes in functional connectivity during waking behavior.

A fundamental operation in neuronal networks is gain modulation, defined as multiplicative or divisive transformations of the neuronal input-output function. Changes in gain amplify or reduce neuronal responsiveness with minimal effects on stimulus selectivity or receptive field structure (Sclar and Freeman, 1982; Carandini and Heeger, 1994). Such a transformation facilitates the interaction among multiple variables in the spiking output of a single neuron. One of the earliest demonstrations of the computational role of gain modulation was the multiplicative increase in visual responses in posterior parietal cortex by eye position (Andersen and Mountcastle, 1983). In addition to implementing sensorimotor transformations, gain modulation has also been shown to play a role in the enhancement of sensory-evoked responses by spatial or feature-based attention (Connor et al., 1996, 1997; McAdams and Maunsell, 1999; Treue and Martinez-Trujillo, 1999; Williford and Maunsell, 2006).

Changes in membrane potential depolarization, membrane potential variance, and membrane conductance, all features of cortical Up states, are ideal candidate mechanisms for rapid gain modulation. Yet, since many basic changes of the neuronal membrane, such as depolarization and shunting, in either model neurons or neurons *in vitro* only effect additive or subtractive shifts in the neuronal input-output function (Holt and Koch, 1997), pure or nearly pure changes in neuronal gain based on fast synaptic signaling (i.e., via ionotropic glutamate and GABA_A_ receptors) have historically been rejected. Many models of gain modulation have relied on more complex mechanisms (Srinivasan and Bernard, 1976; Salinas and Abbott, 1996; Hahnloser et al., 2000). When neurons are embedded in a recurrently active network, however, the presence of synaptic noise fundamentally alters the ability of membrane potential, membrane potential variance, and membrane conductance to modulate gain (Hô and Destexhe, 2000; Chance et al., 2002; Fellous et al., 2003; Mitchell and Silver, 2003; Murphy and Miller, 2003; Shu et al., 2003a; Kuhn et al., 2004). In the presence of synaptic noise, the relation between membrane potential and firing rate is not a linear function with a threshold, as is the case in quiescent networks, but a power law, in which firing rate relates to membrane potential by raising the latter by an exponent (Miller and Troyer, 2002). When the relation between membrane potential and firing rate obeys a power-law function, simple manipulations of the neuronal membrane, such as depolarization and hyperpolarization, can change neuronal gain (Murphy and Miller, 2003). Indeed, such multiplicative modulations due to depolarization alone have been described during Up states in visual cortex *in vivo* (Haider et al., 2007). Thus, enhanced barrages of synaptic inputs, which occur on a rapid time-scale in local cortical networks, could produce immediate changes in neuronal gain in the subset of neurons responsible for momentary behavioral demands. In another model of gain modulation by synaptic inputs, neuronal gain is increased by the *removal* of these inputs (Chance et al., 2002). In this model, EPSPs and IPSPs must be removed in precise proportion to keep membrane potential constant while decreasing membrane conductance. Which model of synaptic-input-dependent gain control is employed by the cortex during active behavior is unclear, but each provides testable predictions: in one model, behaviorally relevant gain control is associated with depolarization, while in the other model, gain control is associated with a decrease in membrane conductance and a constant membrane potential.

## Conclusions

The slow oscillation is a fundamental feature of the corticothalamic system; during behavioral periods associated with effective disconnection from the sensory environment, it is essentially the default activity pattern of the entire cortical mantle. Through the use of various recording and imaging techniques in *in vivo* and *in vitro* preparations, considerable progress has been made in elucidating the cellular and network mechanisms involved in the synchronous cycling of Up and Down states in both local and disparate cortical networks.

There are many further avenues for investigation of the mechanisms and functions of the slow oscillation. Among the most crucial are the causal roles of thalamic activity in the initiation, persistence, and termination of Up states, the causal roles of inhibitory interneurons vs. intrinsic hyperpolarizing conductances in Up state termination, the functions of slow rhythmic activity in quiescent behavioral states, and the precise relation between network activity during the Up state and network activity associated with active sensory and cognitive processing. While these are all quite ambitious questions, the necessary technologies for addressing them at the single-neuron, microcircuit, and large-scale network levels are emerging.

## Author Contribution

GTN wrote the manuscript.

## Conflict of Interest Statement

The author declares that the research was conducted in the absence of any commercial or financial relationships that could be construed as a potential conflict of interest.

## References

[B1] Agmon A. Connors B. W. (1989). Repetitive burst-firing neurons in the deep layers of mouse somatosensory cortex. Neurosci. Lett. 99, 137–141. 10.1016/0304-3940(89)90278-4 2748005

[B2] Agmon A. Connors B. W. (1991). Thalamocortical responses of mouse somatosensory (barrel) cortex *in vitro*. Neuroscience 41, 365–379. 10.1016/0306-4522(91)90333-j 1870696

[B4] Alaburda A. Perrier J. F. Hounsgaard J. (2002). Mechanisms causing plateau potentials in spinal motoneurons. Adv. Exp. Med. Biol. 508, 219–226. 10.1007/978-1-4615-0713-0_27 12171115

[B5] Amzica F. Steriade M. (1995a). Short- and long-range neuronal synchronization of the slow (<1 Hz) cortical oscillation. J. Neurophysiol. 73, 20–38. 7714565 10.1152/jn.1995.73.1.20

[B6] Amzica F. Steriade M. (1995b). Disconnection of intracortical synaptic linkages disrupts synchronization of a slow oscillation. J. Neurosci. 15, 4658–4677. 7790931 10.1523/JNEUROSCI.15-06-04658.1995 PMC6577695

[B7] Amzica F. Steriade M. (1997). The K-complex: its slow (<1-Hz) rhythmicity and relation to delta waves. Neurology 49, 952–959. 10.1212/wnl.49.4.952 9339673

[B8] Andersen R. A. Mountcastle V. B. (1983). The influence of the angle of gaze upon the excitability of the light-sensitive neurons of the posterior parietal cortex. J. Neurosci. 3, 532–548. 6827308 10.1523/JNEUROSCI.03-03-00532.1983 PMC6564545

[B10] Anderson J. Lampl I. Reichova I. Carandini M. Ferster D. (2000). Stimulus dependence of two-state fluctuations of membrane potential in cat visual cortex. Nat. Neurosci. 3, 617–621. 10.1038/75797 10816319

[B11] Arieli A. Sterkin A. Grinvald A. Aertsen A. (1996). Dynamics of ongoing activity: explanation of the large variability in evoked cortical responses. Science 273, 1868–1871. 10.1126/science.273.5283.1868 8791593

[B12] Ashford M. L. Sturgess N. C. Trout N. J. Gardner N. J. Hales C. N. (1988). Adenosine-5′-triphosphate-sensitive ion channels in neonatal rat cultured central neurones. Pflugers. Arch. 412, 297–304. 10.1007/bf00582512 2460821

[B13] Azouz R. Gray C. M. (1999). Cellular mechanisms contributing to response variability of cortical neurons. J. Neurosci. 19, 2209–2223. 10066274 10.1523/JNEUROSCI.19-06-02209.1999 PMC6782570

[B14] Barinaga M. (1994). Neuroscience. To sleep, perchance to…learn? New studies say yes. Science 265, 603–604. 10.1126/science.8036508 8036508

[B15] Barthó P. Freund T. F. Acsády L. (2002). Selective GABAergic innervation of thalamic nuclei from zona incerta. Eur. J. Neurosci. 16, 999–1014. 10.1046/j.1460-9568.2002.02157.x 12383229

[B16] Bazhenov M. Timofeev I. Steriade M. Sejnowski T. J. (2002). Model of thalamocortical slow-wave sleep oscillations and transitions to activated states. J. Neurosci. 22, 8691–8704. 12351744 10.1523/JNEUROSCI.22-19-08691.2002 PMC6757797

[B17] Beierlein M. Gibson J. R. Connors B. W. (2003). Two dynamically distinct inhibitory networks in layer 4 of the neocortex. J. Neurophysiol. 90, 2987–3000. 10.1152/jn.00283.2003 12815025

[B18] Beltramo R. D’Urso G. Dal Maschio M. Farisello P. Bovetti S. Clovis Y.. (2013). Layer-specific excitatory circuits differentially control recurrent network dynamics in the neocortex. Nat. Neurosci. 16, 227–234. 10.1038/nn.3306 23313909

[B19] Binzegger T. Douglas R. J. Martin K. A. C. (2004). A quantitative map of the circuit of cat primary visual cortex. J. Neurosci. 24, 8441–8453. 10.1523/jneurosci.1400-04.2004 15456817 PMC6729898

[B20] Blethyn K. L. Hughes S. W. Tóth T. I. Cope D. W. Crunelli V. (2006). Neuronal basis of the slow (<1 Hz) oscillation in neurons of the nucleus reticularis thalami. J. Neurosci. 26, 2474–2486. 10.1523/jneurosci.3607-05.2006 16510726 PMC6793657

[B21] Borbély A. A. (1980). “Sleep: circadian rhythm versus recovery process,” in Functional States of the Brain: Their Determinants, eds Koukkou M. Lehmann D. Angst J. (Amsterdam: Elsevier), 151–161.

[B22] Bosman C. A. Lansink C. S. Pennartz C. M. (2014). Functional of gamma-band synchronization in cognition: from single circuits to functional diversity across cortical and subcortical systems. Eur. J. Neurosci. 39, 1982–1999. 10.1111/ejn.12606 24809619

[B23] Braitenburg V. Shüz A. (1998). Cortex: Statistics and Geometry of Neuronal Connectivity. New York: Springer.

[B24] Buzsáki G. (2006). Rhythms of the Brain. New York: Oxford University Press.

[B26] Buzsáki G. (2010). Neural syntax: cell assemblies, synapsembles and readers. Neuron 68, 362–385. 10.1016/j.neuron.2010.09.023 21040841 PMC3005627

[B25] Buzsáki G. Leung L. W. Vanderwolf C. H. (1983). Cellular bases of hippocampal EEG in the behaving rat. Brain Res. 287, 139–171. 10.1016/0165-0173(83)90037-1 6357356

[B28] Canolty R. T. Knight R. T. (2010). The functional role of cross-frequency coupling. Trends Cogn. Sci. 14, 506–515. 10.1016/j.tics.2010.09.001 20932795 PMC3359652

[B29] Carandini M. Heeger D. J. (1994). Summation and division by neurons in primate visual cortex. Science 264, 1333–1336. 10.1126/science.8191289 8191289

[B31] Castro-Alamancos M. A. Favero M. (2015). NMDA receptors are the basis for persistent network activity in neocortex slices. J. Neurophysiol. 113, 3816–3826. 10.1152/jn.00090.2015 25878152 PMC4473514

[B32] Chagnac-Amitai Y. Connors B. W. (1989). Synchronized excitation and inhibition driven by intrinsically bursting neurons in neocortex. J. Neurophysiol. 62, 1149–1162. 2585046 10.1152/jn.1989.62.5.1149

[B33] Chagnac-Amitai Y. Luhmann H. J. Prince D. A. (1990). Burst generating and regular spiking layer 5 pyramidal neurons of rat neocortex have different morphological features. J. Comp. Neurol. 296, 598–613. 10.1002/cne.902960407 2358553

[B34] Chance F. S. Abbott L. F. Reyes A. D. (2002). Gain modulation from background synaptic input. Neuron 35, 773–782. 10.1016/S0896-6273(02)00820-6 12194875

[B36] Chauvette S. Crochet S. Volgushev M. Timofeev I. (2011). Properties of slow oscillation during slow-wave sleep and anesthesia in cats. J. Neurosci. 31, 14998–15008. 10.1523/JNEUROSCI.2339-11.2011 22016533 PMC3209581

[B35] Chauvette S. Volgushev M. Timofeev I. (2010). Origin of active states in local neocortical networks during slow sleep oscillation. Cereb. Cortex 20, 2660–2674. 10.1093/cercor/bhq009 20200108 PMC2951844

[B37] Chen J. Y. Chauvette S. Skorheim S. Timofeev I. Bazhenov M. (2012). Interneuron-mediated inhibition synchronizes neuronal activity during slow oscillation. J. Physiol. 590, 3987–4010. 10.1113/jphysiol.2012.227462 22641778 PMC3476644

[B38] Civillico E. F. Contreras D. (2012). Spatiotemporal properties of sensory responses *in vivo* are strongly dependent on network context. Front. Syst. Neurosci. 6:25. 10.3389/fnsys.2012.00025 22509158 PMC3325540

[B39] Compte A. Sanchez-Vives M. V. McCormick D. A. Wang X. J. (2003). Cellular and network mechanisms of slow oscillatory activity (<1 Hz) and wave propagations in a cortical network model. J. Neurophysiol. 89, 2707–2725. 10.1152/jn.00845.2002 12612051

[B40] Connor C. E. Gallant J. L. Preddie D. C. Van Essen D. C. (1996). Responses in area V4 depend on the spatial relationship between stimulus and attention. J. Neurophysiol. 75, 1306–1308. 8867139 10.1152/jn.1996.75.3.1306

[B41] Connor C. E. Preddie D. C. Gallant J. L. Van Essen D. C. (1997). Spatial attention effects in macaque area V4. J. Neurosci. 17, 3201–3214. 9096154 10.1523/JNEUROSCI.17-09-03201.1997 PMC6573654

[B42] Connors B. W. (1984). Initiation of synchronized neuronal bursting in neocortex. Nature 310, 685–687. 10.1038/310685a0 6147755

[B430] Connors B. W. Gutnick M. J. Prince D. A. (1982). Electrophysiological properties of neocortical neurons in vitro. J. Neurophysiol. 48, 1302–1320. 6296328 10.1152/jn.1982.48.6.1302

[B43] Connors B. W. Pinto D. J. Telfeian A. E. (2001). Local pathways of seizure propagation in neocortex. Int. Rev. Neurobiol. 45, 527–546. 10.1016/s0074-7742(01)45027-6 11130915

[B44] Contreras D. Steriade M. (1995). Cellular basis of EEG slow rhythms: a study of dynamic corticothalamic relationships. J. Neurosci. 15, 604–622. 7823167 10.1523/JNEUROSCI.15-01-00604.1995 PMC6578315

[B46] Contreras D. Destexhe A. Sejnowski T. J. Steriade M. (1996a). Control of spatiotemporal coherence of a thalamic oscillation by corticothalamic feedback. Science 274, 771–774. 10.1126/science.274.5288.771 8864114

[B45] Contreras D. Timofeev I. Steriade M. (1996b). Mechanisms of long-lasting hyperpolarizations underlying slow sleep oscillations in cat corticothalamic networks. J. Physiol. 494, 251–264. 10.1113/jphysiol.1996.sp021488 8814619 PMC1160627

[B47] Cortelli P. Gambetti P. Montagna P. Lugaresi E. (1999). Fatal familial insomnia: clinical features and molecular genetics. J. Sleep Res. 8(Suppl. 1), 23–29. 10.1046/j.1365-2869.1999.00005.x 10389103

[B48] Cossart R. Aronov D. Yuste R. (2003). Attractor dynamics of network UP states in the neocortex. Nature 423, 283–288. 10.1038/nature01614 12748641

[B50] Craig M. T. Mayne E. W. Bettler B. Paulsen O. McBain C. (2013). Distinct roles of GABA_B1a_- and GABA_B1b_-containing GABA_B_ receptors in spontaneous and evoked termination of persistent cortical activity. J. Physiol. 591, 835–843. 10.1113/jphysiol.2012.248088 23266934 PMC3591701

[B49] Crochet S. Petersen C. C. (2006). Correlating whisker behavior with membrane potential in barrel cortex of awake mice. Nat. Neurosci. 9, 608–610. 10.1038/nn1690 16617340

[B51] Cruikshank S. J. Rose H. J. Metherate R. (2002). Auditory thalamocortical synaptic transmission *in vitro*. J. Neurophysiol. 87, 361–384. 10.1152/jn.00549.2001 11784756

[B54] Crunelli V. David F. Lörincz M. L. Hughes S. W. (2015). The thalamocortical network as a single slow wave-generating unit. Curr. Opin. Neurobiol. 31, 72–80. 10.1016/j.conb.2014.09.001 25233254

[B53] Crunelli V. Hughes S. W. (2010). The slow (<1 Hz) rhythm of non-REM sleep: a dialogue between three cardinal oscillators. Nat. Neurosci. 13, 9–17. 10.1038/nn.2445 19966841 PMC2980822

[B52] Crunelli V. Lörincz M. L. Errington A. C. Hughes S. W. (2012). Activity of cortical and thalamic neurons during the slow (<1 Hz) rhythm in the mouse. Pflugers. Arch. 463, 73–88. 10.1007/s00424-011-1011-9 21892727 PMC3256325

[B55] Cunningham M. O. Pervouchine D. D. Racca C. Kopell N. J. Davies C. H. Jones R. S. G.. (2006). Neuronal metabolism governs cortical network response state. Proc. Natl. Acad. Sci. U S A 103, 5597–5601. 10.1073/pnas.0600604103 16565217 PMC1459399

[B57] David F. Schmiedt J. T. Taylor H. L. Orban G. Di Giovanni G. Uebele V. N.. (2013). Essential thalamic contribution to slow waves of natural sleep. J. Neurosci. 33, 19599–19610. 10.1523/JNEUROSCI.3169-13.2013 24336724 PMC3858629

[B58] DeFelipe J. Fariñas I. (1992). The pyramidal neuron of the cerebral cortex: morphological and chemical characteristics of the synaptic inputs. Prog. Neurobiol. 39, 563–607. 10.1016/0301-0082(92)90015-7 1410442

[B59] Dembrow N. Johnston D. (2014). Subcircuit-specific neuromodulation in the prefrontal cortex. Front. Neural Circuits 8:54. 10.3389/fncir.2014.00054 24926234 PMC4046580

[B60] Destexhe A. Contreras D. Steriade M. (1999). Spatiotemporal analysis of local field potentials and unit discharges in cat cerebral cortex during natural wake and sleep states. J. Neurosci. 19, 4595–4608. 10341257 10.1523/JNEUROSCI.19-11-04595.1999 PMC6782626

[B61] Destexhe A. Hughes S. W. Rudolph M. Crunelli V. (2007). Are corticothalamic ‘up’ states fragments of wakefulness? Trends Neurosci. 30, 334–342. 10.1016/j.tins.2007.04.006 17481741 PMC3005711

[B62] Détári L. Rasmusson D. D. Semba K. (1997). Phasic relationship between the activity of basal forebrain neurons and cortical EEG in urethane-anesthetized rat. Brain Res. 759, 112–121. 10.1016/s0006-8993(97)00252-7 9219869

[B63] Diekelmann S. Born J. (2010). The memory function of sleep. Nat. Rev. Neurosci. 11, 114–126. 10.1038/nrn2762 20046194

[B64] Douglas R. J. Martin K. A. C. (2004). Neuronal circuits of the neocortex. Annu. Rev. Neurosci. 27, 419–451. 10.1146/annurev.neuro.27.070203.144152 15217339

[B65] Eccles J. C. (1961). “Chairman’s opening remarks,” in CIBA Foundation Symposium on the Nature of Sleep, eds Wolstenholme G. E. W. O’Connor M. (Boston: Little, Brown and Co), 1–3.

[B66] Eggermann E. Kremer Y. Crochet S. Petersen C. C. (2014). Cholinergic signals in mouse barrel cortex during active whisker sensing. Cell Rep. 9, 1654–1660. 10.1016/j.celrep.2014.11.005 25482555

[B67] Egorov A. V. Hamam B. N. Fransén E. Hasselmo M. E. Alonso A. A. (2002). Graded persistent activity in entorhinal cortex neurons. Nature 420, 173–178. 10.1038/nature01171 12432392

[B68] Eschenko O. Magri C. Panzeri S. Sara S. J. (2012). Noradrenergic neurons of the locus coeruleus are phase locked to cortical up-down states during sleep. Cereb. Cortex 22, 426–435. 10.1093/cercor/bhr121 21670101

[B69] Fanselow E. E. Connors B. W. (2010). The roles of somatostatin-expressing (GIN) and fast-spiking inhibitory interneurons in UP-DOWN states of mouse neocortex. J. Neurophysiol. 104, 596–606. 10.1152/jn.00206.2010 20538767 PMC2934925

[B70] Fatt P. Katz B. (1952). Spontaneous subthreshold activity at motor nerve endings. J. Physiol. 117, 109–128. 14946732 PMC1392564

[B72] Favero M. Castro-Alamancos M. A. (2013). Synaptic cooperativity regulates persistent network activity in neocortex. J. Neurosci. 33, 3151–3163. 10.1523/JNEUROSCI.4424-12.2013 23407969 PMC3711603

[B71] Favero M. Varghese G. Castro-Alamancos M. A. (2012). The state of somatosensory cortex during neuromodulation. J. Neurophysiol. 108, 1010–1024. 10.1152/jn.00256.2012 22623484 PMC3424075

[B73] Fellous J. M. Rudolph M. Destexhe A. Sejnowski T. J. (2003). Synaptic background noise controls the input/output characteristic of single cells in an *in vitro* model of *in vivo* activity. Neuroscience 122, 811–829. 10.1016/j.neuroscience.2003.08.027 14622924 PMC2928821

[B74] Flint A. C. Connors B. W. (1996). Two types of network oscillations in neocortex mediated by distinct glutamate receptor subtypes and neuronal populations. J. Neurophysiol. 75, 951–957. 8714667 10.1152/jn.1996.75.2.951

[B75] Frank M. G. Benington J. H. (2006). The role of sleep in memory consolidation and brain plasticity: dream of reality? Neuroscientist 12, 477–488. 10.1177/1073858406293552 17079514

[B76] Frankenhaeuser B. Hodgkin A. L. (1957). The action of calcium on the electrical properties of squid axons. J. Physiol. 137, 218–244. 10.1113/jphysiol.1957.sp005808 13449874 PMC1362975

[B77] Fransén E. Tahvildari B. Egorov A. V. Hasselmo M. E. Alonso A. A. (2006). Mechanism of graded persistent cellular activity of entorhinal cortex layer v neurons. Neuron 49, 735–746. 10.1016/j.neuron.2006.01.036 16504948

[B78] Fries P. (2009). Neuronal gamma-band synchronization as a fundamental process in cortical computation. Annu. Rev. Neurosci. 32, 209–224. 10.1146/annurev.neuro.051508.135603 19400723

[B790] Ghosh A. Greenberg M. E. (1995). Calcium signaling in neurons: molecular mechanisms and cellular consequences. Science 268, 239–247. 10.1126/science.7716515 7716515

[B79] Gibson J. R. Beierlein M. Connors B. W. (1999). Two networks of electrically coupled inhibitory interneurons in neocortex. Nature 402, 75–79. 10.1038/47035 10573419

[B80] Gil Z. Connors B. W. Amitai Y. (1997). Differential regulation of neocortical synapses by neuromodulators and activity. Neuron 19, 679–686. 10.1016/s0896-6273(00)80380-3 9331357

[B81] Goel N. Rao H. Durmer J. S. Dinges D. F. (2009). Neurocognitive consequences of sleep deprivation. Semin. Neurol. 29, 320–339. 10.1055/s-0029-1237117 19742409 PMC3564638

[B82] Golshani P. Liu X. B. Jones E. G. (2001). Differences in quantal amplitude reflect GluR4-subunit number at corticothalamic synapses on two populations of thalamic neurons. Proc. Natl. Acad. Sci. U S A 98, 4172–4177. 10.1073/pnas.061013698 11274440 PMC31198

[B83] Gray C. M. McCormick D. A. (1996). Chattering cells: superficial pyramidal neurons contributing to the generation of synchronous oscillations in the visual cortex. Science 274, 109–113. 10.1126/science.274.5284.109 8810245

[B84] Gutnick M. J. Connors B. W. Prince D. A. (1982). Mechanisms of neocortical epileptogenesis *in vitro*. J. Neurophysiol. 48, 1321–1335. 7153795 10.1152/jn.1982.48.6.1321

[B850] Hahnloser R. H. Sarpeshkar R. Mahowald M. A. Douglas R. J. Seung H. S. (2000). Digital selection and analogue amplification coexist in a cortex-inspired silicon circuit. Nature 405, 947–951. 10.1038/35016072 10879535

[B85] Haider B. Duque A. Hasenstaub A. R. McCormick D. A. (2006). Neocortical network activity *in vivo* is generated through a dynamic balance of excitation and inhibition. J. Neurosci. 26, 4535–4545. 10.1523/jneurosci.5297-05.2006 16641233 PMC6674060

[B86] Haider B. Duque A. Hasenstaub A. R. Yu Y. McCormick D. A. (2007). Enhancement of visual responsiveness by spontaneous local network activity *in vivo*. J. Neurophysiol. 97, 4186–4202. 10.1152/jn.01114.2006 17409168

[B87] Haider B. McCormick D. A. (2009). Rapid neocortical dynamics: cellular and network mechanisms. Neuron 62, 171–189. 10.1016/j.neuron.2009.04.008 19409263 PMC3132648

[B89] Halassa M. M. Chen Z. Wimmer R. D. Brunetti P. M. Zhao S. Zikopoulos B.. (2014). State-dependent architecture of thalamic reticular subnetworks. Cell 158, 808–821. 10.1016/j.cell.2014.06.025 25126786 PMC4205482

[B88] Halassa M. M. Siegle J. H. Ritt J. T. Ting J. T. Feng G. Moore C. I. (2011). Selective optical drive of thalamic reticular nucleus generates thalamic bursts and cortical spindles. Nat. Neurosci. 14, 1118–1120. 10.1038/nn.2880 21785436 PMC4169194

[B91] Hasenstaub A. Sachdev R. N. McCormick D. A. (2007). State changes rapidly modulate cortical responsiveness. J. Neurosci. 27, 9607–9622. 10.1523/jneurosci.2184-07.2007 17804621 PMC6672966

[B90] Hasenstaub A. Shu Y. Haider B. Kraushaar U. Duque A. McCormick D. A. (2005). Inhibitory postsynaptic potentials carry synchronized frequency information in active cortical networks. Neuron 47, 423–435. 10.1016/j.neuron.2005.06.016 16055065

[B92] Hill S. Tononi G. (2005). Modeling sleep and wakefulness in the thalamocortical system. J. Neurophysiol. 93, 1671–1698. 10.1152/jn.00915.2004 15537811

[B95] Hô N. Destexhe A. (2000). Synaptic background activity enhances the responsiveness of neocortical pyramidal neurons. J. Neurophysiol. 84, 1488–1496. 10980021 10.1152/jn.2000.84.3.1488

[B96] Hobson J. A. Pace-Schott E. F. Stickgold R. (2000). Dreaming and the brain: toward a cognitive neuroscience of conscious states. Behav. Brain. Sci. 23, 793–842; discussion 904–1121. 10.1017/S0140525X00003976 11515143

[B97] Holt G. R. Koch C. (1997). Shunting inhibition does not have a divisive effect on firing rates. Neural Comput. 9, 1001–1013. 10.1162/neco.1997.9.5.1001 9188191

[B94] Hsieh C. Y. Cruikshank S. J. Metherate R. (2000). Differential modulation of auditory thalamocortical and intracortical synaptic transmission by cholinergic agonist. Brain Res. 880, 51–64. 10.1016/s0006-8993(00)02766-9 11032989

[B99] Hughes S. W. Cope D. W. Blethyn K. L. Crunelli V. (2002). Cellular mechanisms of the slow (<1 Hz) oscillation in thalamocortical neurons *in vitro*. Neuron 33, 947–958. 10.1016/s0896-6273(02)00623-2 11906700

[B98] Hughes S. W. Cope D. W. Tóth T. I. Williams S. R. Crunelli V. (1999). All thalamocortical neurones possess a T-type Ca^2+^ “window” current that enables the expression of bistability-mediated activities. J. Physiol. 517, 805–815. 10.1111/j.1469-7793.1999.0805s.x 10358120 PMC2269384

[B101] Igelström K. M. (2013). Is slack an intrinsic seizure terminator? Neuroscientist 19, 248–254. 10.1177/1073858412446311 22645110

[B100] Ikegaya Y. Aaron G. Cossart R. Aronov D. Lampl I. Ferster D.. (2004). Synfire chains and cortical songs: temporal modules of cortical activity. Science 304, 559–564. 10.1126/science.1093173 15105494

[B102] Isomura Y. Sirota A. Ozen S. Montgomery S. Mizuseki K. Henze D. A.. (2006). Integration and segregation of activity in entorhinal-hippocampal subregions by neocortical slow oscillations. Neuron 52, 871–882. 10.1016/j.neuron.2006.10.023 17145507

[B103] Ji D. Wilson M. A. (2007). Coordinated memory replay in the visual cortex and hippocampus during sleep. Nat. Neurosci. 10, 100–107. 10.1038/nn1825 17173043

[B104] Jia H. Rochefort N. L. Chen X. Konnerth A. (2010). Dendritic organization of sensory input to cortical neurons *in vivo*. Nature 464, 1307–1312. 10.1038/nature08947 20428163

[B105] Jones E. G. (1998). Viewpoint: the core and matrix of thalamic organization. Neuroscience 85, 331–345. 10.1016/s0306-4522(97)00581-2 9622234

[B106] Jouvet M. (1972). The role of monoamines and acetylcholine-containing neurons in the regulation of the sleep-waking cycle. Ergebn. Physiol. 64, 166–307. 4403272 10.1007/3-540-05462-6_2

[B107] Jouvet M. (1999). Sleep and serotonin: an unfinished story. Neuropsychopharmacology 21, 24S–27S. 10.1038/sj.npp.1395333 10432485

[B108] Karni A. Tanne D. Rubenstein B. S. Askenasy J. J. Sagi D. (1994). Dependence on REM sleep of overnight improvement of a perceptual skill. Science 265, 679–682. 10.1126/science.8036518 8036518

[B109] Killgore W. D. (2010). Effects of sleep deprivation on cognition. Prog. Brain Res. 185, 105–129. 10.1016/b978-0-444-53702-7.00007-5 21075236

[B110] Kim U. Bal T. McCormick D. A. (1995). Spindle waves are propagating synchronized oscillations in the ferret LGNd *in vitro*. J. Neurophysiol. 74, 1301–1323. 7500152 10.1152/jn.1995.74.3.1301

[B111] Krishnamurthy P. Silberberg G. Lansner A. (2012). A cortical attractor network with Martinotti cells driven by facilitating synapses. PLoS One 7:e30752. 10.1371/journal.pone.0030752 22523533 PMC3327695

[B112] Kulik A. Vida I. Luján R. Haas C. A. López-Bendito G. Shigemoto R.. (2003). Subcellular localization of metabotropic GABA_B_ receptor subunits GABA_B1a/b_ and GABA_B2_ in the rat hippocampus. J. Neurosci. 23, 11026–11035. 14657159 10.1523/JNEUROSCI.23-35-11026.2003 PMC6741037

[B113] Kuhn A. Aertsen A. Rotter S. (2004). Neuronal integration of synaptic input in the fluctuation-driven regime. J. Neurosci. 24, 2345–2356. 10.1523/jneurosci.3349-03.2004 15014109 PMC6729484

[B114] Le Bon-Jego M. Yuste R. (2007). Persistently active, pacemaker-like neurons in neocortex. Front. Neurosci. 1, 123–129. 10.3389/neuro.01.1.1.009.2007 18982123 PMC2518052

[B116] Lemieux M. Chauvette S. Timofeev I. (2015). Neocortical inhibitory activities and long-range afferents contribute to the synchronous onset of silent states of the neocortical slow oscillation. J. Neurophysiol. 113, 768–779. 10.1152/jn.00858.2013 25392176 PMC4312868

[B115] Lemieux M. Chen J. Y. Lonjers P. Bazhenov M. Timofeev I. (2014). The impact of cortical deafferentation on the neocortical slow oscillation. J. Neurosci. 34, 5689–5703. 10.1523/JNEUROSCI.1156-13.2014 24741059 PMC3988418

[B117] Lisman J. E. Fellous J. M. Wang X. J. (1998). A role for NMDA-receptor channels in working memory. Nat. Neurosci. 1, 273–275. 10.1038/1086 10195158

[B119] Llinás R. R. Grace A. A. Yarom Y. (1991). *In vitro* neurons in mammalian cortical layer 4 exhibit intrinsic oscillatory activity in the 10-to 50-Hz frequency range. Proc. Natl. Acad. Sci. U S A 88, 897–901. 10.1073/pnas.88.8.3510-c 1992481 PMC50921

[B120] Llinás R. Ribary U. (1993). Coherent 40-Hz oscillation characterizes dream state in humans. Proc. Natl. Acad. Sci. U S A 90, 2078–2081. 10.1073/pnas.90.5.2078 8446632 PMC46024

[B118] Llinás R. Sugimori M. (1980). Electrophysiological properties of *in vitro* Purkinje cell dendrites in mammalian cerebellar slices. J. Physiol. 305, 197–213. 10.1113/jphysiol.1980.sp013358 7441553 PMC1282967

[B121] Loewenstein Y. Mahon S. Chadderton P. Kitamura K. Sompolinsky H. Yarom Y.. (2005). Bistability of cerebellar Purkinje cells modulated by sensory stimulation. Nat. Neurosci. 8, 202–211. 10.1038/nn1393 15665875

[B122] Lörincz M. L. Gunner D. Bao Y. Connelly W. M. Isaac J. T. R. Hughes S. W.. (2015). A distinct class of slow (~0.2–2 Hz) intrinsically bursting layer 5 pyramidal neurons determines UP/DOWN state dynamics in the neocortex. J. Neurosci. 35, 5442–5458. 10.1523/JNEUROSCI.3603-14.2015 25855163 PMC4388913

[B123] Luczak A. Barthó P. Marguet S. L. Buzsáki G. Harris K. D. (2007). Sequential structure of neocortical spontaneous activity. Proc. Natl. Acad. Sci. U S A 104, 347–352. 10.1073/pnas.0605643104 17185420 PMC1765463

[B124] MacLean J. N. Watson B. O. Aaron G. B. Yuste R. (2005). Internal dynamics determine the cortical response to thalamic stimulation. Neuron 48, 811–823. 10.1016/j.neuron.2005.09.035 16337918

[B126] Major G. Larkum M. E. Schiller J. (2013). Active properties of neocortical pyramidal neuron dendrites. Annu. Rev. Neurosci. 36, 1–24. 10.1146/annurev-neuro-062111-150343 23841837

[B125] Major G. Tank D. (2004). Persistent neural activity: prevalence and mechanisms. Curr. Opin. Neurobiol. 14, 675–684. 10.1016/j.conb.2004.10.017 15582368

[B127] Mann E. O. Kohl M. M. Paulsen O. (2009). Distinct roles of GABA_A_ and GABA_B_ receptors in balancing and terminating persistent cortical activity. J. Neurosci. 29, 7513–7518. 10.1523/JNEUROSCI.6162-08.2009 19515919 PMC4326656

[B128] Manns I. D. Alonso A. Jones B. E. (2000). Discharge properties of juxtacellularly labeled and immunohistochemically identified cholinergic basal forebrain neurons recorded in association with electroencephalogram in anesthetized rats. J. Neurosci. 20, 1505–1518. 10662840 10.1523/JNEUROSCI.20-04-01505.2000 PMC6772366

[B129] Mao B. Q. Hamzei-Sichani F. Aronov D. Froemke R. C. Yuste R. (2001). Dynamics of spontaneous activity in neocortical slices. Neuron 32, 883–898. 10.1016/s0896-6273(01)00518-9 11738033

[B130] Marder E. Abbott L. F. Turrigiano G. G. Liu Z. Golowasch J. (1996). Memory from the dynamics of intrinsic currents. Proc. Natl. Acad. Sci. U S A 93, 13481–13486. 10.1073/pnas.93.24.13481 8942960 PMC33634

[B132] Massimini M. Ferrarelli F. Huber R. Esser S. K. Singh H. Tononi G. (2005). Breakdown of cortical effective connectivity during sleep. Science 309, 2228–2232. 10.1126/science.1117256 16195466

[B131] Massimini M. Huber R. Ferrarelli F. Hill S. Tononi G. (2004). The sleep slow oscillation as a traveling wave. J. Neurosci. 24, 6862–6870. 10.1523/jneurosci.1318-04.2004 15295020 PMC6729597

[B133] Massimini M. Tononi G. Huber R. (2009). Slow waves, synaptic plasticity and information processing: insights from transcranial magnetic stimulation and high-density EEG experiments. Eur. J. Neurosci. 29, 1761–1770. 10.1111/j.1460-9568.2009.06720.x 19473231 PMC2776746

[B134] Mayne E. W. Craig M. T. McBain C. J. Paulsen O. (2013). Dopamine suppresses persistent network activity via D(1)-like dopamine receptors in rat medial entorhinal cortex. Eur. J. Neurosci. 37, 1242–1247. 10.1111/ejn.12125 23336973 PMC3628042

[B135] McAdams C. J. Maunsell J. H. (1999). Effects of attention on orientation-tuning functions of single neurons in macaque cortical area V4. J. Neurosci. 19, 431–441. 9870971 10.1523/JNEUROSCI.19-01-00431.1999 PMC6782389

[B138] McCormick D. A. (1992). Neurotransmitter actions in the thalamus and cerebral cortex and their role in neuromodulation of thalamocortical activity. Prog. Neurobiol. 39, 337–388. 10.1016/0301-0082(92)90012-4 1354387

[B142] McCormick D. A. Contreras D. (2001). On the cellular and network bases of epileptic seizures. Annu. Rev. Physiol. 63, 815–846. 10.1146/annurev.physiol.63.1.815 11181977

[B140] McCormick D. A. Pape H. C. (1990). Properties of a hyperpolarization-activated cation current and its role in rhythmic oscillation in thalamic relay neurons. J. Physiol. 431, 291–318. 10.1113/jphysiol.1990.sp018331 1712843 PMC1181775

[B144] McCormick D. A. Shu Y. Hasenstaub A. (2004). “Balanced recurrent excitation and inhibition in local cortical networks,” in Excitatory-Inhibitory Balance: Synapses, Circuits, Systems, eds Hensch T. K. Fagiolini M. (New York: Kluwer), 113–122.

[B143] McCormick D. A. Shu Y. Hasenstaub A. Sanchez-Vives M. Badoual M. Bal T. (2003). Persistent cortical activity: mechanisms of generation and effects on neuronal excitability. Cereb. Cortex 13, 1219–1231. 10.1093/cercor/bhg104 14576213

[B141] McCormick D. A. von Krosigk M. (1992). Corticothalamic activation modulates thalamic firing through glutamate “metabotropic” receptors. Proc. Natl. Acad. Sci. U S A 89, 2774–2778. 10.1073/pnas.89.7.2774 1313567 PMC48745

[B137] McCormick D. A. Wang Z. (1991). Serotonin and noradrenaline excite GABAergic neurones of the guinea-pig and cat nucleus reticularis thalami. J. Physiol. 442, 235–255. 10.1113/jphysiol.1991.sp018791 1665858 PMC1179887

[B139] McCormick D. A. Williamson A. (1989). Convergence and divergence of neurotransmitter action in human cerebral cortex. Proc. Natl. Acad. Sci. U S A 86, 8098–8102. 10.1073/pnas.86.20.8098 2573061 PMC298222

[B145] McCormick D. A. Yuste R. (2006). “UP states and cortical dynamics,” in Microcircuits: the Interface between Neurons and Global Brain Function, eds Grillner S. Graybiel A. M. (Cambridge: MIT Press), 327–346.

[B146] McCoy J. G. Strecker R. E. (2011). The cognitive cost of sleep lost. Neurobiol. Learn. Mem. 96, 564–582. 10.1016/j.nlm.2011.07.004 21875679 PMC3614362

[B147] McGinty D. J. Harper R. M. (1976). Dorsal raphe neurons: depression of firing during sleep in cats. Brain Res. 101, 569–575. 10.1016/0006-8993(76)90480-7 1244990

[B136] McLaughlin S. G. Szabo G. Eisenman G. (1971). Divalent ions and the surface potential of charged phospholipid membranes. J. Gen. Physiol. 58, 667–687. 10.1085/jgp.58.6.667 5120393 PMC2226047

[B148] McNamara P. Johnson P. McLaren D. Harris E. Beauharnais C. Auerbach S. (2010). REM and NREM sleep mentation. Int. Rev. Neurobiol. 92, 69–86. 10.1016/S0074-7742(10)92004-7 20870063

[B149] Melamed O. Barak O. Silberberg G. Markram H. Tsodyks M. (2008). Slow oscillations in neural networks with facilitating synapses. J. Comput. Neurosci. 25, 308–316. 10.1007/s10827-008-0080-z 18483841

[B151] Mena-Segovia J. Sims H. M. Magill P. J. Bolam J. P. (2008). Cholinergic brainstem neurons modulate cortical gamma activity during slow oscillations. J. Physiol. 586, 2947–2960. 10.1113/jphysiol.2008.153874 18440991 PMC2517196

[B150] Metherate R. Cruikshank S. J. (1999). Thalamocortical inputs trigger a propagating envelope of gamma-band activity in auditory cortex. Exp. Brain Res. 126, 160–174. 10.1007/s002210050726 10369139

[B152] Miller K. D. Troyer T. W. (2002). Neural noise can explain expansive, power-law nonlinearities in neural response functions. J. Neurophysiol. 87, 653–659. 10.1152/jn.00425.2001 11826034

[B153] Mitchell S. J. Silver R. A. (2003). Shunting inhibition modulates neuronal gain during synaptic excitation. Neuron 38, 433–445. 10.1016/s0896-6273(03)00200-9 12741990

[B154] Mochol G. Hermoso-Mendizabal A. Sakata S. Harris K. D. de la Rocha J. (2015). Stochastic transitions into silence cause noise correlations in cortical circuits. Proc. Natl. Acad. Sci. U S A 112, 3529–3534. 10.1073/pnas.1410509112 25739962 PMC4371940

[B155] Monckton J. E. McCormick D. A. (2002). Neuromodulatory role of serotonin in the ferret thalamus. J. Neurophysiol. 87, 2124–2136. 10.1152/jn.00650.2001 11929930

[B157] Moruzzi G. (1966). “The functional significance of sleep with particular regard to the brain mechanisms underlying consciousness,” in Brain and Conscious Experience, ed. Eccles J. C. (New York: Springer), 345–379.

[B156] Moruzzi G. Magoun H. W. (1949). Brain stem reticular formation and activation of the EEG. Electroencephalogr. Clin. Neurophysiol. 1, 455–473. 10.1016/0013-4694(49)90219-9 18421835

[B159] Muñoz W. Rudy B. (2014). Spatiotemporal specificity in cholinergic control of neocortical function. Curr. Opin. Neurobiol. 26, 149–160. 10.1016/j.conb.2014.02.015 24637201 PMC4100208

[B158] Murphy B. K. Miller K. D. (2003). Multiplicative gain changes are induced by excitation or inhibition alone. J. Neurosci. 23, 10040–10051. 14602818 10.1523/JNEUROSCI.23-31-10040.2003 PMC6740852

[B160] Neske G. T. Patrick S. L. Connors B. W. (2015). Contributions of diverse excitatory and inhibitory neurons to recurrent network activity in cerebral cortex. J. Neurosci. 35, 1089–1105. 10.1523/JNEUROSCI.2279-14.2015 25609625 PMC4300319

[B161] Nuñez A. Amzica F. Steriade M. (1992). Voltage-dependent fast (20–40 Hz) oscillations in long-axoned neocortical neurons. Neuroscience 51, 7–10. 10.1016/0306-4522(92)90464-d 1465188

[B162] Packer A. M. Roska B. Häusser M. (2013). Targeting neurons and photons for optogenetics. Nat. Neurosci. 16, 805–815. 10.1038/nn.3427 23799473 PMC4928704

[B163] Paré D. Shink E. Gaudreau H. Destexhe A. Lang E. J. (1998). Impact of spontaneous synaptic activity on the resting properties of cat neocortical pyramidal neurons *in vivo*. J. Neurophysiol. 79, 1450–1460. 9497424 10.1152/jn.1998.79.3.1450

[B164] Pavlov I. P. (1923). Address on “the identity of inhibition with hypnosis and sleep.” Q. J. Exp. Physiol. 13, 39–43. 10.1113/expphysiol.1923.sp000307

[B165] Petersen C. H. Hahn T. T. G. Mehta M. Grinvald A. Sakmann B. (2003). Interaction of sensory responses with spontaneous depolarization in layer 2/3 barrel cortex. Proc. Natl. Acad. Sci. U S A 100, 13638–13643. 10.1073/pnas.2235811100 14595013 PMC263866

[B166] Phillis J. W. Kostopoulos G. K. Limacher J. J. (1975). A potent depressant action of adenine derivatives on cerebral cortical neurons. Eur. J. Pharmacol. 30, 125–129. 10.1016/0014-2999(75)90214-9 164352

[B167] Pinto D. J. Patrick S. L. Huang W. C. Connors B. W. (2005). Initiation, propagation and termination of epileptiform activity in rodent neocortex *in vitro* involve distinct mechanisms. J. Neurosci. 25, 8131–8140. 10.1523/jneurosci.2278-05.2005 16148221 PMC6725540

[B168] Poulet J. F. Petersen C. C. (2008). Internal brain state regulates membrane potential synchrony in barrel cortex of behaving mice. Nature 454, 881–885. 10.1038/nature07150 18633351

[B170] Pritchett D. L. Siegle J. H. Deister C. A. Moore C. I. (2015). For things needing your attention: the role of neocortical gamma in sensory perception. Curr. Opin. Neurobiol. 31, 254–263. 10.1016/j.conb.2015.02.004 25770854

[B171] Puig M. V. Ushimaru M. Kawaguchi Y. (2008). Two distinct activity patterns of fast-spiking interneurons during neocortical UP states. Proc. Natl. Acad. Sci. U S A 105, 8428–8433. 10.1073/pnas.0712219105 18550841 PMC2448853

[B172] Ramirez D. M. Kavalali E. T. (2011). Differential regulation of spontaneous and evoked neurotransmitter release at central synapses. Curr. Opin. Neurobiol. 21, 275–282. 10.1016/j.conb.2011.01.007 21334193 PMC3092808

[B173] Reig R. Gallego R. Nowak L. G. Sanchez-Vives M. V. (2006). Impact of cortical network activity on short-term synaptic depression. Cereb. Cortex 16, 688–695. 10.1093/cercor/bhj014 16107589

[B174] Reig R. Zerlaut Y. Vergara R. Destexhe A. Sanchez-Vives M. V. (2015). Gain modulation of synaptic inputs by network state in auditory cortex *in vivo*. J. Neurosci. 35, 2689–2702. 10.1523/JNEUROSCI.2004-14.2015 25673859 PMC6605611

[B175] Reyes A. Lujan R. Rozov A. Burnashev N. Somogyi P. Sakmann B. (1998). Target-cell-specific facilitation and depression in neocortical circuits. Nat. Neurosci. 1, 279–285. 10.1038/1092 10195160

[B177] Rigas P. Castro-Alamancos M. A. (2007). Thalamocortical Up states: differential effects of intrinsic and extrinsic cortical inputs on persistent activity. J. Neurosci. 27, 4261–4272. 10.1523/jneurosci.0003-07.2007 17442810 PMC6672324

[B178] Rigas P. Castro-Alamancos M. A. (2009). Impact of persistent cortical activity (Up states) on intracortical and thalamocortical synaptic inputs. J. Neurophysiol. 102, 119–131. 10.1152/jn.00126.2009 19403750 PMC2712261

[B179] Ros H. Sachdev R. N. Yu Y. Sestan N. McCormick D. A. (2009). Neocortical networks entrain neuronal circuits in cerebellar cortex. J. Neurosci. 29, 10309–10320. 10.1523/JNEUROSCI.2327-09.2009 19692605 PMC3137973

[B180] Rudolph M. Pospischil M. Timofeev I. Destexhe A. (2007). Inhibition determines membrane potential dynamics and controls action potential generation in awake and sleeping cat cortex. J. Neurosci. 27, 5280–5290. 10.1523/jneurosci.4652-06.2007 17507551 PMC6672346

[B181] Sachdev R. N. S. Ebner F. F. Wilson C. J. (2004). Effect of subthreshold up and down states on the whisker-evoked response in somatosensory cortex. J. Neurophysiol. 92, 3511–3521. 10.1152/jn.00347.2004 15254074

[B182] Sakai K. Crochet S. (2001). Differentiation of presumed serotonergic dorsal raphe neurons in relation to behavior and wake-sleep states. Neuroscience 104, 1141–1155. 10.1016/s0306-4522(01)00103-8 11457597

[B183] Sakata S. Harris K. D. (2009). Laminar structure of spontaneous and sensory-evoked population activity in auditory cortex. Neuron 64, 404–418. 10.1016/j.neuron.2009.09.020 19914188 PMC2778614

[B184] Salinas E. Abbott L. F. (1996). A model of multiplicative neural responses in parietal cortex. Proc. Natl. Acad. Sci. U S A 93, 11956–11961. 10.1073/pnas.93.21.11956 8876244 PMC38165

[B185] Salkoff D. B. Zagha E. Yüzgeç Ö. McCormick D. A. (2015). Synaptic mechanisms of tight spike synchrony at gamma frequency in cerebral cortex. J. Neurosci. 35, 10236–10251. 10.1523/JNEUROSCI.0828-15.2015 26180200 PMC4502264

[B186] Sallanon M. Buda C. Janin M. Jouvet M. (1983). Serotonergic mechanisms and sleep rebound. Brain Res. 268, 95–104. 10.1016/0006-8993(83)90393-1 6222783

[B190] Sanchez-Vives M. V. (2007). “An active cortical network *in vitro*,” in Mechanisms of Spontaneous Active States in the Neocortex, ed. Timofeev I. (Kerala, India: Research Signpost), 23–44.

[B191] Sanchez-Vives M. V. Descalzo V. F. Reig R. Figueroa N. A. Compte A. Gallego R. (2008). Rhythmic spontaneous activity in the piriform cortex. Cereb. Cortex 18, 1179–1192. 10.1093/cercor/bhm152 17925296

[B192] Sanchez-Vives M. V. Mattia M. Compte A. Perez-Zabalza M. Winograd M. Descalzo V. F.. (2010). Inhibitory modulation of cortical up states. J. Neurophysiol. 104, 1314–1324. 10.1152/jn.00178.2010 20554835

[B188] Sanchez-Vives M. V. McCormick D. A. (2000). Cellular and network mechanisms of rhythmic recurrent activity in neocortex. Nat. Neurosci. 3, 1027–1034. 10.1038/79848 11017176

[B189] Sanchez-Vives M. V. Nowak L. G. McCormick D. A. (2000). Cellular mechanisms of long-lasting adaptation in visual cortical neurons. J. Neurosci. 20, 4286–4299. 10818164 10.1523/JNEUROSCI.20-11-04286.2000 PMC6772630

[B193] Schweimer J. V. Mallet N. Sharp T. Ungless M. A. (2011). Spike-timing relationship of neurochemically-identified dorsal raphe neurons during cortical slow oscillations. Neuroscience 196, 115–123. 10.1016/j.neuroscience.2011.08.072 21925244 PMC3235546

[B194] Schwindt P. C. Spain W. J. Crill W. E. (1989). Long-lasting reduction of excitability by a sodium-dependent potassium current in cat neocortical neurons. J. Neurophysiol. 61, 233–244. 2918352 10.1152/jn.1989.61.2.233

[B195] Schwindt P. C. Spain W. J. Crill W. E. (1992). Calcium-dependent potassium currents in neurons from cat sensorimotor cortex. J. Neurophysiol. 67, 216–226. 1313080 10.1152/jn.1992.67.1.216

[B1960] Sclar G. Freeman R. D. (1982). Orientation selectivity in the cat’s striate cortex is invariant with stimulus contrast. Exp. Brain Res. 46, 457–461. 10.1007/BF00238641 7095050

[B196] Sheroziya M. Timofeev I. (2014). Global intracellular slow-wave dynamics of the thalamocortical system. J. Neurosci. 34, 8875–8893. 10.1523/JNEUROSCI.4460-13.2014 24966387 PMC4069359

[B197] Shu Y. Hasenstaub A. Badoual M. Bal T. McCormick D. A. (2003a). Barrages of synaptic activity control the gain and sensitivity of cortical neurons. J. Neurosci. 23, 10388–10401. 14614098 10.1523/JNEUROSCI.23-32-10388.2003 PMC6741011

[B198] Shu Y. Hasenstaub A. McCormick D. A. (2003b). Turning on and off recurrent balanced cortical activity. Nature 423, 288–293. 10.1038/nature01616 12748642

[B199] Siapas A. G. Wilson M. A. (1998). Coordinated interactions between hippocampal ripples and cortical spindles during slow-wave sleep. Neuron 21, 1123–1128. 10.1016/s0896-6273(00)80629-7 9856467

[B200] Silberberg G. Markram H. (2007). Disynaptic inhibition between neocortical pyramidal cells mediated by Martinotti cells. Neuron 53, 735–746. 10.1016/j.neuron.2007.02.012 17329212

[B201] Silva L. R. Amitai Y. Connors B. W. (1991). Intrinsic oscillations of neocortex generated by layer 5 pyramidal neurons. Science 251, 432–435. 10.1126/science.1824881 1824881

[B202] Sippy T. Yuste R. (2013). Decorrelating action of inhibition in neocortical networks. J. Neurosci. 33, 9813–9830. 10.1523/JNEUROSCI.4579-12.2013 23739978 PMC3715137

[B204] Sirota A. Buzsáki G. (2005). Interaction between neocortical hippocampal networks via slow oscillations. Thalamus Relat. Syst. 3, 245–259. 10.1017/s1472928807000258 18185848 PMC2180396

[B203] Sirota A. Csicsvari J. Buhl D. Buzsáki G. (2003). Communication between neocortex and hippocampus during sleep in rodents. Proc. Natl. Acad. Sci. U S A 100, 2065–2069. 10.1073/pnas.0437938100 12576550 PMC149959

[B2050] Soderling T. R. Derkach V. A. (2000). Postsynaptic protein phosphorylation and LTP. Trends Neurosci. 23, 75–80. 10.1016/S0166-2236(99)01490-3 10652548

[B205] Somjen G. G. (2004). Ions in the Brain: Normal Function, Seizures and Stroke. Oxford: Oxford University Press.

[B206] Srinivasan M. V. Bernard G. D. (1976). A proposed mechanism for the multiplication of neural signals. Biol. Cybern. 21, 227–236. 10.1007/bf00344168 174752

[B220] Steriade M. Amzica F. (1996). Intracortical and corticothalamic coherency of fast spontaneous oscillations. Proc. Natl. Acad. Sci. U S A 93, 2533–2538. 10.1073/pnas.93.6.2533 8637909 PMC39832

[B221] Steriade M. Amzica F. Contreras D. (1996a). Synchronization of fast (30–40 Hz) spontaneous cortical rhythms during brain activation. J. Neurosci. 16, 392–417. 8613806 10.1523/JNEUROSCI.16-01-00392.1996 PMC6578724

[B222] Steriade M. Contreras D. Amzica F. Timofeev I. (1996b). Synchronization of fast (30–40 Hz) spontaneous oscillations in intrathalamic and thalamocortical networks. J. Neurosci. 16, 2788–2808. 8786454 10.1523/JNEUROSCI.16-08-02788.1996 PMC6578775

[B219] Steriade M. Contreras D. Amzica F. (1994). Synchronized sleep oscillations and their paroxysmal developments. Trends Neurosci. 17, 199–208. 10.1016/0166-2236(94)90105-8 7520202

[B210] Steriade M. Domich L. Oakson G. Deschênes M. (1987). The deafferented reticularis thalami nucleus generates spindle rhythmicity. J. Neurophysiol. 57, 260–273. 3559675 10.1152/jn.1987.57.1.260

[B212] Steriade M. Dossio R. C. Paré D. Oakson G. (1991a). Fast oscillations (20–40 Hz) in thalamocortical systems and their potentiation by mesopontine cholinergic nuclei in the cat. Proc. Natl. Acad. Sci. U S A 88, 4396–4400. 10.1073/pnas.88.10.4396 2034679 PMC51666

[B213] Steriade M. Dossi R. C. Nuñez A. (1991b). Network modulation of a slow intrinsic oscillation of cat thalamocortical neurons implicated in sleep delta waves: cortical potentiation and brainstem cholinergic suppression. J. Neurosci. 11, 3200–3217. 1941080 10.1523/JNEUROSCI.11-10-03200.1991 PMC6575431

[B214] Steriade M. Nuñez A. Amzica F. (1993a). A novel (<1 Hz) oscillation of neocortical neurons *in vivo*: depolarizing and hyperpolarizing components. J. Neurosci. 13, 3252–3265. 8340806 10.1523/JNEUROSCI.13-08-03252.1993 PMC6576541

[B215] Steriade M. Nuñez A. Amzica F. (1993b). Intracellular analysis of relations between the slow (<1 Hz) neocortical oscillation and other sleep rhythms of the electroencephalogram. J. Neurosci. 13, 3266–3283. 8340807 10.1523/JNEUROSCI.13-08-03266.1993 PMC6576520

[B216] Steriade M. Contreras D. Curró Dossi R. Nuñez A. (1993c). The slow (<1 Hz) oscillation in reticular thalamic and thalamocortical neurons: scenario of sleep rhythm generation in interacting thalamic and neocortical networks. J. Neurosci. 13, 3284–3299. 8340808 10.1523/JNEUROSCI.13-08-03284.1993 PMC6576531

[B217] Steriade M. Amzica F. Nuñez A. (1993d). Cholinergic and noradrenergic modulation of the slow (~0.3 Hz) oscillation in neocortical cells. J. Neurophysiol. 70, 1385–1400. 8283204 10.1152/jn.1993.70.4.1385

[B223] Steriade M. Timofeev I. Grenier F. (2001). Natural waking and sleep states: a view from inside neocortical neurons. J. Neurophysiol. 85, 1969–1985. 11353014 10.1152/jn.2001.85.5.1969

[B224] Steriade M. Timofeev I. (2003). Neuronal plasticity in thalamocortical networks during sleep and waking oscillations. Neuron 37, 563–576. 10.1016/s0896-6273(03)00065-5 12597855

[B225] Stickgold R. (2005). Sleep-dependent memory consolidation. Nature 437, 1272–1278. 10.1038/nature04286 16251952

[B226] Tahvildari B. Wölfel M. Duque A. McCormick D. A. (2012). Selective functional interactions between excitatory and inhibitory cortical neurons and differential contribution to persistent activity of the slow oscillation. J. Neurosci. 32, 12165–12179. 10.1523/JNEUROSCI.1181-12.2012 22933799 PMC3466092

[B227] Telfeian A. E. Connors B. W. (1998). Layer-specific pathways for the horizontal propagation of epileptiform discharges in neocortex. Epilepsia 39, 700–708. 10.1111/j.1528-1157.1998.tb01154.x 9670897

[B228] Thomson A. M. Deuchars J. (1994). Temporal and spatial properties of local circuits in neocortex. Trends Neurosci. 17, 119–126. 10.1016/0166-2236(94)90121-x 7515528

[B230] Timofeev I. Contreras D. Steriade M. (1996). Synaptic responsiveness of cortical and thalamic neurones during various phases of slow sleep oscillation in cat. J. Physiol. 494, 265–278. 10.1113/jphysiol.1996.sp021489 8814620 PMC1160628

[B231] Timofeev I. Grenier F. Bazhenov M. Sejnowski T. J. Steriade M. (2000). Origin of slow cortical oscillations in deafferented cortical slabs. Cereb. Cortex 10, 1185–1199. 10.1093/cercor/10.12.1185 11073868

[B229] Timofeev I. Steriade M. (1996). Low-frequency rhythms in the thalamus of intact-cortex and decorticated cats. J. Neurophysiol. 76, 4152–4168. 8985908 10.1152/jn.1996.76.6.4152

[B232] Tononi G. Cirelli C. (2014). Sleep and the price of plasticity: from synaptic and cellular homeostasis to memory consolidation and integration. Neuron 81, 12–34. 10.1016/j.neuron.2013.12.025 24411729 PMC3921176

[B233] Treue S. Martinez-Trujillo J. C. (1999). Feature-based attention influences motion processing gain in macaque visual cortex. Nature 399, 575–579. 10.1038/21176 10376597

[B234] Trulson M. E. Jacobs B. L. (1979). Raphe unit activity in freely moving cats: correlation with level of behavioral arousal. Brain Res. 163, 135–150. 10.1016/0006-8993(79)90157-4 218676

[B235] Van Dongen H. P. Belenky G. Krueger J. M. (2011). A local, bottom-up perspective on sleep deprivation and neurobehavioral performance. Curr. Top. Med. Chem. 11, 2414–2422. 10.2174/156802611797470286 21906023 PMC3243827

[B236] Vertes R. P. (2004). Memory consolidation in sleep: dream or reality. Neuron 44, 135–148. 10.1016/j.neuron.2004.08.034 15450166

[B237] Volgushev M. Chauvette S. Mukovski M. Timofeev I. (2006). Precise long-range synchronization of activity and silence during slow-wave sleep. J. Neurosci. 26, 5665–5672. 10.1523/jneurosci.0279-06.2006 16723523 PMC6675259

[B239] Vyazovskiy V. V. Harris K. D. (2013). Sleep and the single neuron: the role of global slow oscillations in individual cell rest. Nat. Rev. Neurosci. 14, 443–451. 10.1038/nrn3494 23635871 PMC3972489

[B238] Vyazovskiy V. V. Olcese U. Hanlon E. C. Nir Y. Cirelli C. Tononi G. (2011). Local sleep in awake rats. Nature 472, 443–447. 10.1038/nature10009 21525926 PMC3085007

[B240] Walker M. P. Stickgold R. (2004). Sleep-dependent learning and memory consolidation. Neuron 44, 121–133. 10.1016/j.neuron.2004.08.031 15450165

[B242] Wang Y. Neubauer F. B. Lüscher H. R. Thurley K. (2010). GABA_B_ receptor-dependent modulation of network activity in the rat prefrontal cortex *in vitro*. Eur. J. Neurosci. 31, 1582–1594. 10.1111/j.1460-9568.2010.07191.x 20525071

[B241] Wang M. Yang Y. Wang C. J. Gamo N. J. Jin L. E. Mazer J. A.. (2013). NMDA receptors subserve persistent neuronal firing during working memory in dorsolateral prefrontal cortex. Neuron 77, 736–749. 10.1016/j.neuron.2012.12.032 23439125 PMC3584418

[B243] Waters J. Helmchen F. (2006). Background synaptic activity is sparse in neocortex. J. Neurosci. 26, 8267–8277. 10.1523/jneurosci.2152-06.2006 16899721 PMC6673816

[B244] Watson B. O. MacLean J. N. Yuste R. (2008). UP states protect ongoing cortical activity from thalamic inputs. PLoS One 3:e3971. 10.1371/journal.pone.0003971 19092994 PMC2597736

[B245] Wester J. C. Contreras D. (2012). Columnar interactions determine horizontal propagation of recurrent network activity in neocortex. J. Neurosci. 32, 5454–5471. 10.1523/JNEUROSCI.5006-11.2012 22514308 PMC3415278

[B246] Wester J. C. Contreras D. (2013). Differential modulation of spontaneous and evoked thalamocortical network activity by acetylcholine level *in vitro*. J. Neurosci. 33, 17951–17966. 10.1523/JNEUROSCI.1644-13.2013 24198382 PMC3818561

[B247] Williams S. R. Turner J. P. Tóth T. I. Hughes S. W. Crunelli V. (1997). The “window” component of the low threshold Ca^2+^ current produces input signal amplification and bistability in cat and rat thalamocortical neurones. J. Physiol. 505, 689–705. 10.1111/j.1469-7793.1997.689ba.x 9457646 PMC1160046

[B248] Williford T. Maunsell J. H. (2006). Effects of spatial attention on contrast response functions in macaque area V4. J. Neurophysiol. 96, 40–54. 10.1152/jn.01207.2005 16772516

[B249] Wilson C. J. Groves P. M. (1981). Spontaneous firing patterns of identified spiny neurons in the rat neostriatum. Brain Res. 220, 67–80. 10.1016/0006-8993(81)90211-0 6168334

[B250] Wilson M. A. McNaughton B. L. (1994). Reactivation of hippocampal ensemble memories during sleep. Science 265, 676–679. 10.1126/science.8036517 8036517

[B2520] Yassin L. Benedetti B. L. Jouhanneau J. S. Wen J. A. Poulet J. F. A. Barth A. L. (2010). An embedded subnetwork of highly active neurons in the neocortex. Neuron 68, 1043–1050. 10.1016/j.neuron.2010.11.029 21172607 PMC3022325

[B251] Yuste R. Tank D. W. (1996). Dendritic integration in mammalian neurons, a century after Cajal. Neuron 16, 701–716. 10.1016/s0896-6273(00)80091-4 8607989

[B252] Žiburkus J. Cressman J. R. Schiff S. J. (2013). Seizures as imbalanced up states: excitatory and inhibitory conductances during seizure-like events. J. Neurophysiol. 109, 1296–1306. 10.1152/jn.00232.2012 23221405 PMC3602838

